# An integrated lipidomics and metabolomics reveal nephroprotective effect and biochemical mechanism of Rheum officinale in chronic renal failure

**DOI:** 10.1038/srep22151

**Published:** 2016-02-23

**Authors:** Zhi-Hao Zhang, Nosratola D. Vaziri, Feng Wei, Xian-Long Cheng, Xu Bai, Ying-Yong Zhao

**Affiliations:** 1 Key Laboratory of Resource Biology and Biotechnology in Western China, Ministry of Education, the College of Life Sciences, Northwest University, No. 229 Taibai North Road, Xi’an, Shaanxi 710069, China; 2 Division of Nephrology and Hypertension, School of Medicine, University of California, Irvine, MedSci 1, C352, UCI Campus, Irvine, California, 92697, USA; 3 National Center for Natural Products Research, Department of BioMolecular Sciences, School of Pharmacy, University of Mississippi, Oxford, Mississippi, 38677, USA; 4 National Institutes for Food and Drug Control, State Food and Drug Administration, No. 2 Tiantan Xili, Beijing, 100050, China; 5 Solution Centre, Waters Technologies Ltd., No. 1000 Jinhai Road, Shanghai 201203, China

## Abstract

Chronic renal failure (CRF) is a major public health problem worldwide. Earlier studies have revealed salutary effects of rhubarb extracts in CRF. In this study, we employed lipidomic and metabolomic approaches to identify the plasma biomarkers and to determine the effect of treatment with petroleum ether, ethyl acetate and n-butanol extracts of rhubarb in a rat model of CRF with adenine-induced chronic tubulointerstitial nephropathy. In addition, clinical biochemistry, histological evaluation and pro-fibrotic protein expression were analyzed. Significant changes were found between the CRF and control groups representing characteristic phenotypes of rats with CRF. Treatment with the three rhubarb extracts improved renal injury and dysfunction, either fully or partially reversed the plasma metabolites abnormalities and attenuated upregulation of pro-fibrotic proteins including TGF-β1, α-SMA, PAI-1, CTGF, FN and collagen-1. The nephroprotective effect of ethyl acetate extract was better than other extracts. The differential metabolites were closely associated with glycerophospholipid, fatty acid and amino acid metabolisms. The results revealed a strong link between renal tubulointerstitial fibrosis and glycerophospholipid metabolism and L-carnitine metabolism in the development of CRF. Amelioration of CRF with the three rhubarb extracts was associated with the delayed development and/or reversal the disorders in key metabolites associated with adenine-induced CRF.

Chronic renal failure (CRF) is a major public health problem worldwide. Progression of CRF is associated with interstitial fibrosis which is characterized by monocyte and macrophage infiltration, tubular atrophy and fibroblast proliferation/differentiation, events that cause extracellular matrix accumulation and tubulointerstitial fibrosis^1^. Therefore therapeutic interventions aimed at prevention, attenuation or reversal of interstitial fibrosis can be highly effective in retarding progression of CRF. Unfortunately, effective anti-fibrotic treatment remains elusive.

Natural medicines have been used for the treatment of different diseases for thousands of years. Anti-fibrotic natural medicines have gained increasing attention for treatment of CRF based on their long-standing and widespread clinical application in the oriental societies. Unfortunately, due to the limited scientific data on the nature and mechanisms of their actions, the therapeutic effect of natural medicines has not been completely recognized. Rhubarb, a well-known natural medicine, possesses various pharmacological properties such as diuretic, nephroprotective, lipid lowering, purgative and anti-inflammatory activities^2^,^3^,^4^. Rhubarb is officially listed in Chinese, Japanese and European Pharmacopoeias. Clinically, rhubarb is commonly used as an ingredient of Chinese herbal formula for the treatment of CRF. Several studies have suggested favorable effect of the treatment with rhubarb in humans and animals with CRF^5^,^6^,^7^,^8^. Although the clinical efficacy of rhubarb in the treatment of CRF has been demonstrated, the underlying biochemical mechanisms of its effect in CRF remain uncertain.

Multiple components of natural medicines can simultaneously hit multiple targets involved in the pathogenesis of the diseases. Metabolomic approach reveals the global metabolic networks which reflect the pathophysiological states in humans and animals and as such can serve as a valuable tool in understanding the mechanisms and clinical diagnosis of the disease and its response to the therapeutic interventions^9^. An increasing number of publications have described metabolomic studies of CRF using various techniques including proton nuclear magnetic resonance and liquid chromatography coupled with mass spectrometry (LC−MS)^10^,^11^,^12^. Earlier studies have revealed numerous lipid abnormalities in patients and animals with CRF^13^,^14^,^15^. However, most of these studies have focused on plasma level and metabolism of lipids and lipoproteins^16^,^17^. Lipidomics, as a branch of metabolomics, is an analytical approach to holistic investigation of multi-parametric response of living systems based on the lipid metabolic profiles in the complex biological samples^18^. Analysis of the key lipid species has become an important part of the disease diagnosis, disease prognosis, drug discovery and drug toxicity evaluation^19^,^20^,^21^. Lipidomic studies have identified characteristic lipid signatures that have potential as diagnostic tools. Modern lipidomics depends almost entirely on analysis by mass spectrometry and chromatography techniques which have greatly promoted the developments and applications of lipidomics^22^,^23^, as it can identify a wide variety of individual lipid species in several classes. Among those analytical techniques, ultra-performance liquid chromatography coupled with quadrupole time-of-flight synapt high-definition mass spectrometry (UPLC-QTOF/HDMS) is most suitable for lipidomics, especially for untargeted lipid profiles due to its enhanced reproducibility of retention time^24^,^25^.

The present study was undertaken to explore the abnormities of lipid metabolism and related metabolites in CRF. To this end, we employed a UPLC-QTOF/HDMS-based plasma lipidomic and metabolomic approaches to identify the metabolic profile, potential biomarkers and the response to treatment with three different rhubarb extracts including petroleum ether extract (PE), ethyl acetate extract (EA) and n-butanol extract (BU) in rats with adenine-induced CRF.

## Results

### General data

**Figure 1:**
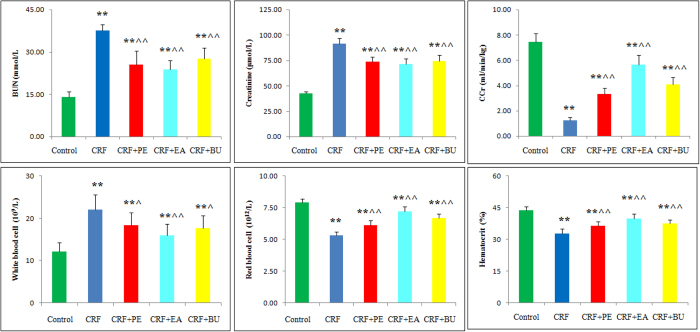
Biochemical parameters in the control, CRF, CRF + PE, CRF + EA and CRF + BU groups. Plasma creatinine, BUN, Ccr, white blood cell, red blood cell and hematocrit in the control, CRF, CRF + PE, CRF + EA and CRF + BU groups. *p < 0.05, **p < 0.01 compared to control group; ^p < 0.05, ^^p < 0.01 compared to CRF group.

Plasma creatinine and blood urea Nitrogen (BUN) concentrations, and white blood cell counts were significantly increased whereas creatinine clearance (Ccr), red blood cell count and hematocrit were significantly decreased in CRF group compared to the control group (Fig. 1). Compared with the CRF group, the EA + CRF group showed significantly lower creatinine and BUN levels and white blood cell counts and significantly higher Ccr, red blood cell count and hematocrit level. The PE + CRF group showed significant decrease in BUN and creatinine concentrations and white blood cell count and significant increase in Ccr, hematocrit and red blood cell count. The BU + CRF group showed significant decrease in BUN, creatinine and white blood cell count and significant increase in Ccr, hematocrit and red blood cell count.

### Renal histology data

**Figure 2:**
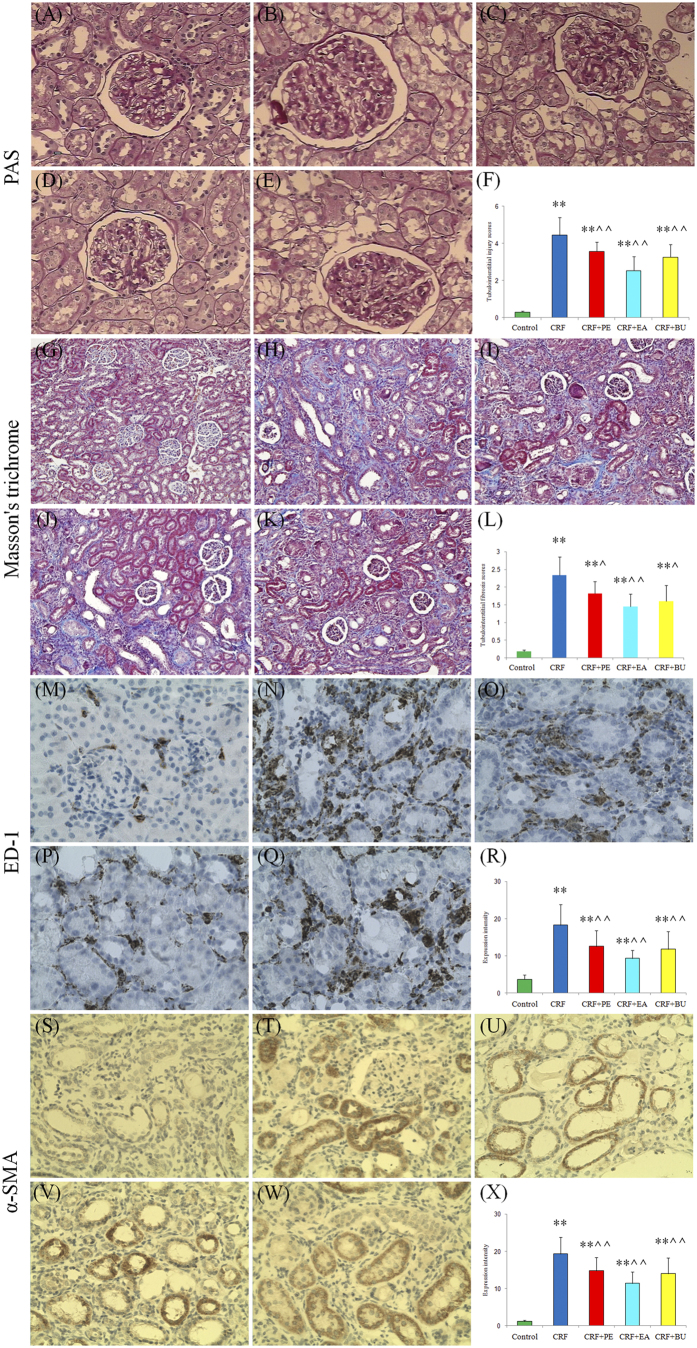
Representative photomicrographs of the PAS staining, Masson’s trichrome staining, ED-1 and α-SMA immunohistochemistry from kidney sections in control, CRF, CRF + PE, CRF + EA and CRF + BU groups. Bar graphs depict renal tubulointerstitial injury scores (**F**), renal tubulointerstitial fibrosis scores (**X**) as well as expression intensity of ED-1 (**L**) and α-SMA immunohistochemistry (**R**) in the study groups. (**A,G,M**,**S**) control group; (**B,H,N**,**T**) CRF group; (**C,I,O**,**U**) CRF + PE group; (**D,J,P**,**V**) CRF + EA group; (**E,K,Q**,**W**) CRF + BU group. *p < 0.05, **p < 0.01 compared to control group; **^**p < 0.05, **^^**p < 0.01 compared to CRF group.

Figure 2 shows representative photomicrographs of periodic acid Schiff (PAS) and Masson’s trichrome stained sections of the kidney tissues. The kidney tissues in rats with CRF showed severe renal tubulointerstitial injury marked by tubular atrophy and dilation, severe interstitial inflammatory cell infiltration and fibrosis. The severity of renal tubulointerstitial injury in rats with CRF was significantly improved by the treatment with PE, EA and BU extracts.

Macrophages play an important role in the development of tubulointerstitial inflammation. ED-1 staining was used to reveal changes in macrophage infiltration in kidney tissues. Compared with the control rats, the numbers of ED-1-positive cells was significantly increased in the interstitium of the rats with CRF and was significantly reduced by the treatment with PE, EA and BU extracts.

### Fibrosis pathway proteins

**Figure 3:**
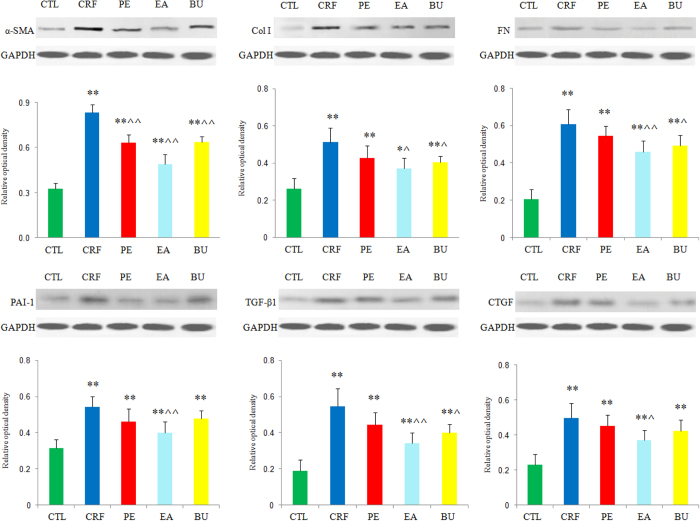
Renal fibrotic protein expression in the five different groups. Expression levels of α-SMA, collagen I, FN, PAI-1, TGF-β1 and CTGF proteins were determined in control, CRF, CRF + PE, CRF + EA and CRF + BU groups by Western blot analysis. Bar graphs depicted expression intensity of α-SMA, collagen I, FN, PAI-1, TGF-β1 and CTGF proteins. GAPDH served as the loading control. *p < 0.05, **p < 0.01 compared to control group; **^**p < 0.05, **^^**p < 0.01 compared to CRF group.

Alpha smooth muscle actin (α-SMA), is a characteristic protein of smooth muscle cells and myofibroblasts, and its high expression in kidney promotes trans-differentiation of tubular epithelial cells to myofibroblasts. As shown in Fig. 2M–R, very low level of α-SMA was detected in kidneys of control rats. In contrast, α-SMA was extensively expressed in renal tubules of the rats with CRF and its expression was attenuated by treatment with PE, EA and BU extracts. The presence of severe renal interstitial fibrosis in rats with CRF was accompanied by the elevated expression of renal fibrotic proteins including α-SMA, plasminogen activator inhibitor-1 (PAI-1), transforming growth factor beta 1 (TGF-β1), connective tissue growth factor (CTGF), fibronectin (FN) and collagen-1 (Fig. 3). Compared with the CRF group, expressions of all of the above protein were significantly attenuated in EA + CRF group; α-SMA expression was significantly reduced in PE + CRF group; α-SMA, collagen I, FN and TGF-β1 expression was significantly lowered in BU + CRF group. Taken together the data revealed that tubulointerstitial injury is substantially ameliorated by treatment with PE, EA and BU extracts. Based on the results of the clinical chemistry, histological analysis and protein expression, nephroprotective effect of EA extract was greater than BU and PE extracts.

### Validation of UPLC−MS conditions

One plasma sample for metabolomic analysis was chosen as the QC sample, and the extracted ion chromatographic peaks of eight ions (3.29_237.1132, 0.55_114.0663, 0.54_162.1124, 0.59_267.059, 3.29_195.1015, 7.20_335.2211, 0.44_110.0087, 1.63_130.0646) were selected for method validation. One plasma sample for lipidomic analysis was also chosen as the QC sample, and the extracted ion chromatographic peaks of four positive ions (8.08_894.8487, 4.67_786.5963, 0.44_337.2097, 8.10_932.871) and four negative ions (3.57_826.5606, 3.63_861.5519, 0.59_433.2354, 1.80_255.2316) were selected for method validation. Reproducibility of the UPLC-MS data was determined from six replicated analyses of the same plasma sample. The RSD (relative standard deviations) of retention time, peak area and m/z of the plasma sample for metabolomic analysis were below 0.54%, 2.8% and 0.00051–0.0027%, respectively. The RSD of retention time, peak area and m/z of the plasma sample for lipidomic analysis were below 0.55%, 3.0% and 0.00045–0.0022%, respectively. The post-preparation stability of samples was tested by analyzing two QC samples kept in autosampler at 4 °C for 4, 8, 12 and 24 h. The RSD of peak areas of the plasma sample for lipidomic and metabolomicanalyses were 2.9% to 6.3% and 2.7% to 7.0%, respectively. The recovery rate (%) for LPC (15:0) and PC (36:1) were 86.50 and 96.22 respectively.

### Metabolic variation and biomarker identification

**Figure 4:**
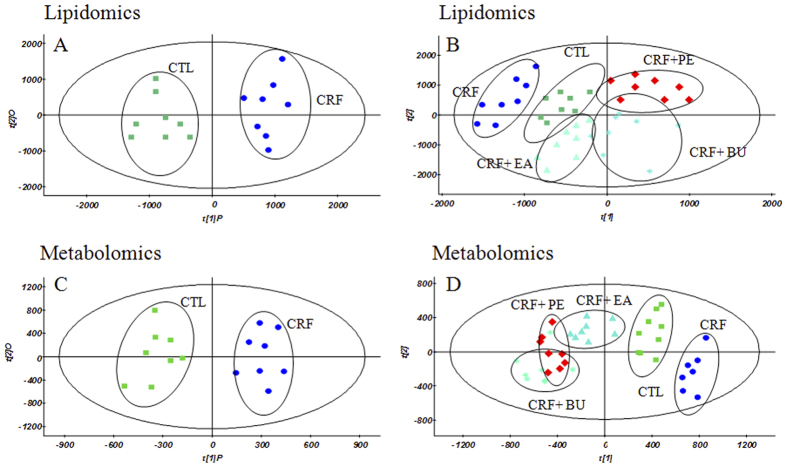
OPLS-DA score plots of the lipidomics (**A**) and metabolomics (**C**) of comparing control group and CRF group; PLS-DA score plots of the lipidomics (**B**) and metabolomics (**D**) of comparing control, CRF, CRF + PE, CRF + EA and CRF + BU groups. The model parameters were: (**A**) *R*^2^*X* = 0.507, *R*^2^*Y* = 0.979, *Q*^2^ = 0.661; (**B**) *R*^2^*X* = 0.839, *R*^2^*Y* = 0.984, *Q*^2^ = 0.584; and (**C**) *R*^2^*X* = 0.513, *R*^2^*Y* = 0.963, *Q*^2^ = 0.855; (**D**) *R*^2^*X* = 0.671, *R*^2^*Y* = 0.978, *Q*^2^ = 0.746.

Lipidomic profile of plasma sample was acquired using UPLC-QTOF/HDMS in ESI^+^ and ESI^−^ modes. Since lipids detected in both ESI^+^ and ESI^−^ modes complemented each other, data obtained from ESI^+^ and ESI^−^ modes were combined to perform statistical analysis. Metabolomic profile of plasma sample was acquired in ESI^+^ mode. Principal component analysis (PCA) was performed on the dataset, which showed a trend of intergroup separation on the scores plot (figures not provided). Orthogonal partial least square discriminant analysis (OPLS-DA) was built to exhibit the metabolic distinction between the control and CRF groups from lipidomics (Fig. 4A) and metabolomics (Fig. 4C). Partial least-square-discriminant analysis (PLS-DA) was built to exhibit the metabolic distinction among control, CRF, CRF + PE, CRF + EA and CRF + BU groups from lipidomics (Fig. 4B) and metabolomics (Fig. 4D). The OPLS-DA and PLS-DA score plots showed good fitness and high predictability of model with high statistical values of R^2^ and Q^2^ (Fig. 4). In the OPLS-DA score plot, metabolic profile of the control and CRF groups was separated from each other clearly (Fig. 4A,C), suggesting remarkable alteration of plasma metabolites in the CRF model. In addition, CRF + PE, CRF + EA and CRF + BU groups affected the metabolite variations in different directions and PLS-DA score plot showed a clear separation between these three groups and the CRF group (Fig. 4B,D). However, as shown in Fig. 4B,D, CRF + EA group is closer to control group than CRF + PE and CRF + BU groups, indicating CRF + EA group had a better therapeutic effect than that observed in the CRF + PE and CRF + BU groups.

**Figure 5:**
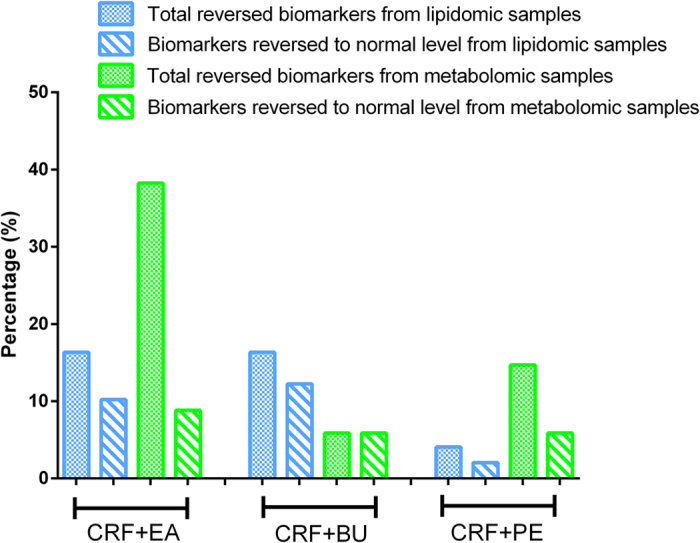
Percentage of the reversed biomarkers in the total biomarkers from lipidomics and metabolomics; Percentage of the biomarkers that reversed to normal level in the total biomarkers from lipidomics and metabolomics. This figure reflected the therapeutic effect of PE, EA and BU treatments on CRF rats.

**Figure 6:**
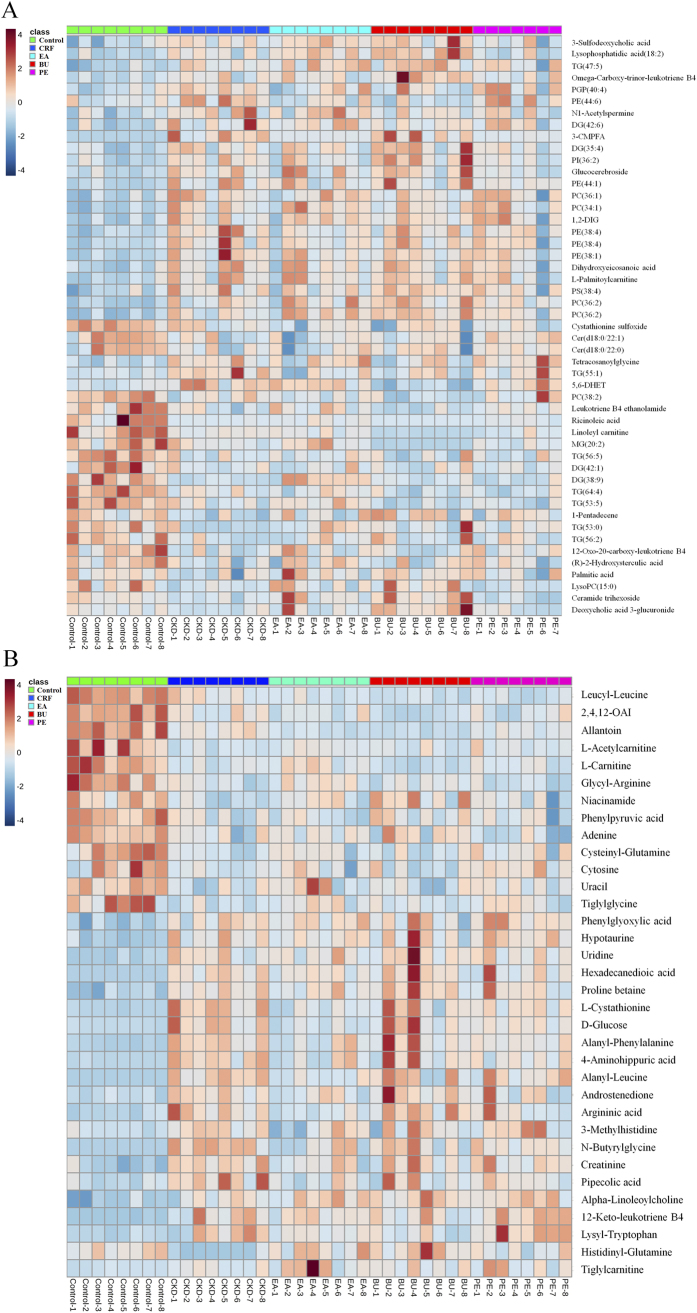
Heat maps of significant metabolites between control and CRF groups from lipidomics (**A**) and metabolomics (**B**). The color of each section is proportional to the significance of change of metabolites (red, up-regulated; blue, down-regulated). 1,2-DIG: 1,2-Di-(9Z,12Z,15Z-octadecatrienoyl)-3-(galactosyl-alpha-1-6- galactosyl-beta-1)-glycerol; 2,4,12-OAI: 2,4,12-Octadecatrienoic acid isobutylamide. Rows: metabolites; Columns: samples.

A total of 83 differential metabolites were identified according to the previously reported method^26^,^27^. Briefly, structure identification was performed according to their molecular ion masses, MS^E^ fragments and i-FIT value comparing with authentic standards or literatures and database resources. Compared to the control group, 49 lipids and 34 metabolites were significantly altered in CRF group from lipidomics and metabolomics, respectively (Tables 1 and 2). They were selected by using the VIP (variable importance in the projection) values (>1.0) combined with Student’s t test (p < 0.05) with a false discovery rate (FDR) <0.05 and Mann-Whitney U test (p < 0.05). In addition, we analyzed intensity changes of the 83 metabolites between CRF + EA and control groups, CRF + EA and CRF groups, CRF + BU and control groups, CRF + BU and CRF groups, CRF + PE and control groups, CRF + PE and CRF groups, respectively (Tables S1 and S2). The results showed that EA, BU and PE extracts all possessed certain therapeutic effect by normalizing or partially reversing the adenine-induced alterations. The changes of the metabolites corresponded with the changes of clinical biochemistry, histology and protein expression. The aim of metabolomics was to investigate the plasma metabolic profile to identify potential biomarkers of CRF and to further understand the nephroprotective effect of three rhubarb extracts. Likewise lipidomics was applied to focus on lipids that related to CRF and the nephroprotective effect of three rhubarb extracts. As shown in Fig. 5, amelioration of CRF by EA, BU and PE treatments were accompanied by the reversal of the abnormalities of the plasma small molecular metabolites as well as lipid metabolites. Furthermore, EA treatment showed better therapeutic effect than BU and PE treatments from metabolomics. For lipidomics, EA and BU treatments showed almost the same therapeutic effect, which was better than PE treatment. In addition, heatmap showed direct variation of each differential metabolite. Figure 6A,B presents the differential metabolites revealing the relatively up-regulated (red) or down-regulated (blue) intensities of the lipidomic and metabolomic compounds in CRF compared with the control group. As shown in Fig. 6, control and CRF groups could be clearly separated based on 49 lipid metabolites as well as 34 small molecular metabolites. Moreover, EA, BU and PE treatments improved metabolic alterations in CRF group by influencing multiple metabolic pathways.

**Figure 7:**
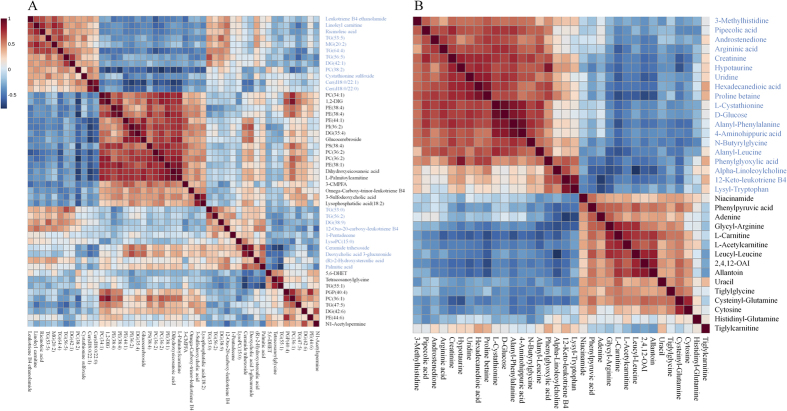
Hierarchical clustering of differential metabolites. (**A**) Correlation analysis of 14 differential metabolites in control and CRF groups from lipidomics. (**B**) Correlation analysis of 15 differential metabolites in control and CRF groups from metabolomics.

Based on the results depicted in Tables S1 and S2, fourteen and fifteen reversed differential metabolites from lipidomics and metabolomics were selected as biomarkers of nephroprotective effects of different rhubarb extracts against CRF, respectively. To narrow down the scope of the biomarker pool, two approaches were combined and employed to further select specific biomarkers and to clearly characterize the nephroprotective effect of EA, BU and PE extracts. First, we used hierarchical cluster analysis to reveal the potential relationships among lipid metabolites. The differential lipid metabolites were divided into four major clusters based on their Pearson correlation coefficients, which were presented in the plot with different colors (Fig. 7A). Cluster 1 consisted of PEs and 5,6-DHET. Cluster 2 was glucocerebroside, TG(55:1) and 3-carboxy-4-methyl-5-propyl-2-furanpropionic acid (3-CMPFA). Cluster 3 contains LPC(15:0) and fatty acids. Cluster 4 was TG(53:0) and DG(38:9). The result indicated that the similar type lipid metabolites were distributed in the same cluster and had similar changing trends. On the other hand, small molecular metabolites were divided into two major clusters based on their Pearson correlation coefficients, which were presented in the plot with different colors (Fig. 7B). Cluster 1 mainly consisted of D-glucose, creatinine and amino acids. Cluster 2 was mainly carnitines and amino acids.

**Figure 8:**
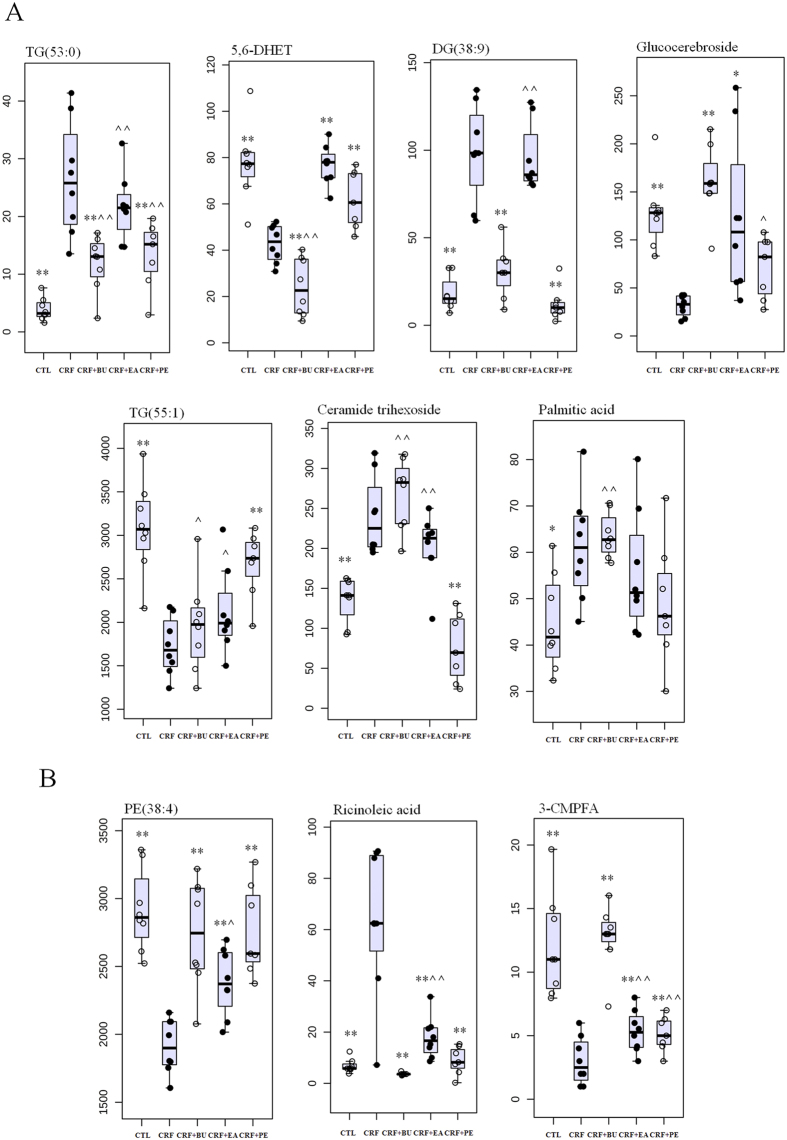
Combined box-and-whisker and dot plot of relative abundance of 10 identified biomarkers from lipidomics in control (CTL), CRF, CRF + PE, CRF + EA and CRF + BU groups. The statistical significance of differences between the two groups was marked. *p < 0.05, **p < 0.01 compared to control group; **^**p < 0.05, **^^**p < 0.01 compared to CRF group.

**Figure 9:**
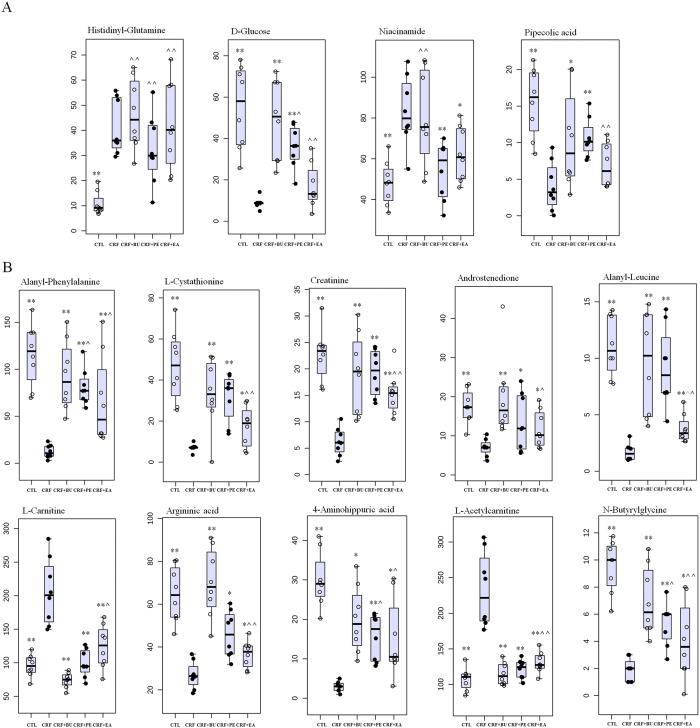
Combined box-and-whisker and dot plot of relative abundance of 14 identified biomarkers from metabolomics in control (CTL), CRF, CRF + PE, CRF + EA and CRF + BU groups. The statistical significance of differences between the two groups was marked. *p < 0.05, **p < 0.01 compared to control group; **^**p < 0.05, **^^**p < 0.01 compared to CRF group.

Next, PLS-DA-based receiver operating characteristic (ROC) curve was performed on each metabolite to further find specific biomarkers. The area under the curve (AUC), 95% confidence interval (95%CI), sensitivity and specificity were shown in Figures S1 and S2. The result showed that 10 out of 14 differential metabolites from lipidomics achieved an AUC of more than 0.85. They had high sensitivity and specificity for validation of the biomarkers including TG(53:0), 5,6-DHET, DG(38:9), ricinoleic acid, glucocerebroside, TG(55:1), ceramide trihexoside, PE(38:4), 3-CMPFA and palmitic acid. For metabolomics, 14 out of 15 differential metabolites achieved an AUC of more than 0.85. They also had high sensitivity and specificity for validation of the biomarkers including alanyl-phenylalanine, L-cystathionine, creatinine, androstenedione, histidinyl-glutamine, L-carnitine, D-glucose, argininic acid, niacinamide, 4-aminohippuric acid, L-acetylcarnitine, pipecolic acid, N-butyrylglycine and alanyl-leucine. Seven out of ten representative biomarkers from lipidomics were completely reversed to normal level by the treatment with PE, EA and BU extracts (Fig. 8A). Three out of the ten representative biomarkers from lipidomics were significantly altered by the treatment with EA, BU or PE compared to the CRF group (Fig. 8B). Four out of fourteen representative biomarkers from metabolomics were completely reversed to normal level by the treatment with PE, EA and BU extracts (Fig. 9A). Treatment with EA, BU or PE resulted in significant changes in ten out of fourteen representative biomarkers from metabolomics in CRF (Fig. 9B). Based on the above results, these 24 metabolites including TG(53:0), 5,6-DHET, DG(38:9), ricinoleic acid, glucocerebroside, TG(55:1), ceramide trihexoside, PE(38:4), 3-CMPFA, palmitic acid, alanyl-phenylalanine, L-cystathionine, creatinine, androstenedione, histidinyl-glutamine, L-carnitine, D-glucose, argininic acid, niacinamide, 4-aminohippuric acid, L-acetylcarnitine, pipecolic acid, N-butyrylglycine and alanyl-leucine were selected as the biomarkers of CRF and also as biomarkers of anti-fibrotic treatment with rhubarb, which were associated with the biochemical mechanism of rhubarb anti-fibrotic activity.

## Discussion

Although the therapeutic efficacy of rhubarb for the treatment of CRF has been demonstrated, the biochemical mechanism of the anti-fibrotic action is not well understood. Due to the limitation of traditional research method and analytical technology, available information on the metabolites involved in the biochemical mechanism of action of rhubarb and its multiple components and multiple targets have been limited. Therefore, the present UPLC-QTOF/HDMS-based lipidomics and metabolomics was performed to obtain a global view of the alterations of plasma metabolome and lipidome and to discover potential biomarkers of adenine-induced CRF and its response to treatment with different rhubarb extracts.

**Figure 10:**
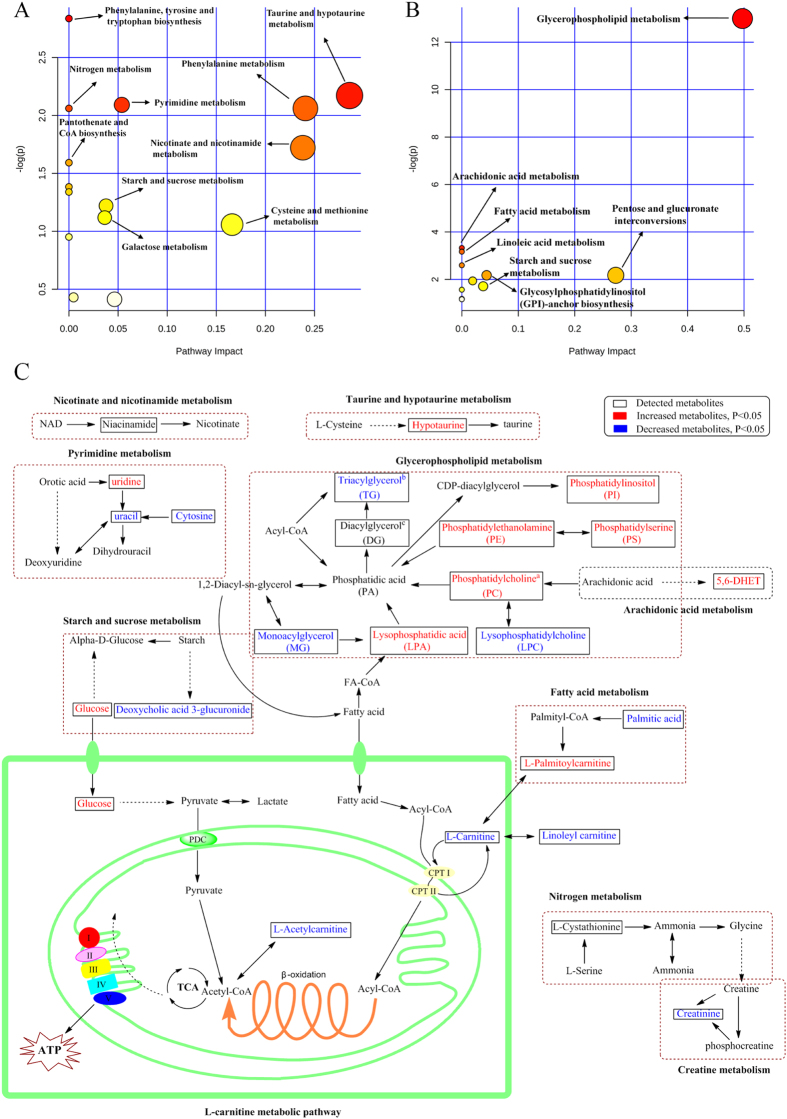
Pathway analysis of the identified metabolites. (**A**) Pathway analysis of 49 differential metabolites from lipidomics. (**B**) Pathway analysis of 34 differential metabolites from metabolomics. Global metabolic disorders of the most relevant pathways induced by adenine were revealed using the MetaboAnalyst; small p value and big pathway impact factor indicate that the pathway is greatly influenced. (**C**) Metabolic pathway of identified metabolites: taurine and hypotaurine metabolism, pyrimidine metabolism, arachidonic acid metabolism, starch and sucrose metabolism, fatty acid metabolism and L-carnitine metabolism. Red color represents increased metabolites in CRF group; Blue color represents decreased metabolites in CRF group. Dotted arrow indicates multiple processes. Solid arrow indicates single process. ^a^PC(36/2), PC(34:1), PC(36:1) and PC(36:2) were significantly increased in CRF group; PC(38:2) was significantly decreased in CRF group. ^b^TG(56:5), TG(64:4), TG(53:0), TG(53:5) and TG(56:2) were significantly decreased in CRF group; TG(47:5) and TG(55:1) were significantly increased in CRF group. ^c^DG(42:1) and DG(38:9) were significantly decreased in CRF group; DG(35:4) and DG(42:6) were significantly increased in CRF group.

To have a better understanding of the biomarkers, we performed a systematic pathway analysis of the lipidome and metabolome. The CRF-associated metabolic perturbation was analyzed from the perspective of pathway enrichment analysis. The most relevant pathways on the basis of the 49 differential metabolites from lipidomics are shown in Fig. 10A and those related to the 34 differential metabolites from metabolomics are shown in Fig. 10B. The data revealed profound perturbations of the metabolisms of glycerophospholipid, fatty acids, L-carnitine, pyrimidine and arachidonic acid in rats with adenine-induced CRF. Furthermore, treatment with EA, BU and PE extracts ameliorated renal lesions, improved kidney function and plasma metabolite abnormalities in rats with adenine-induced CRF. Particularly, treatment with EA was more effective than BU and PE in restoring the abnormal metabolites.

Lipids are the fundamental components of biological membranes. Cumulative evidence has suggested that abnormality in lipid metabolism contributes to the progression of renal disease^28^,^29^,^30^. Phospholipids have been reported to be markers for chronic glomerulonephritis, IgA nephropathy, chronic renal failure and diabetic nephropathy^31^. The identified metabolites in rats with CRF included seven triacylglycerols (TG), four diacylglycerols (DG), one monoacylglycerol (MG), five phosphatidylcholines (PC), one lysophosphatidylcholine (LPC), one lysophosphatidic acid (LPA), five phosphatidylethanolamines (PE), one phosphatidylserine (PS) and one phosphatidylinositol (PI). The marked alteration of these lipid metabolites in CRF group points to disturbance of the metabolic pathway of glycerophospholipid (Fig. 10C).

PC is synthesized through the cytidine diphosphate-choline or kennedy pathway. Alternatively, it can be synthesized by the direct methylation of the ethanolamine residue of PE catalyzed by phosphatidylethanolamine N-methyltransferase. PC is hydrolyzed by phospholipase A2, phosphatidylcholine-phospholipase C and phospholipase D to release LPC, DG, phosphatidic acid and free fatty acids. The increase in PC(36:2), PC(34:1), PC(36:1), PC(36:2), PE(38:4), PE(44:1), PE(38:1), PE(38:4), DG(42:6) and DG(35:4) observed in rats with adenine-induced CRF is in agreement with a previous report on the levels of PC in POKO mice which exhibit rapid progression of renal disease with marked increase in the inflammatory and pro-fibrotic markers^32^. LPCs are products of PCs which are structural components of animal cell membranes. The rats with adenine-induced CRF employed in our study exhibited marked down-regulation of LPC(15:0). The observed reduction in LPC might be due to accumulation of uremic toxins/metabolites that inhibit the activity of phospholipase A2 and lead to the reduction of LPC generation^33^.

LPA is a growth factor-like phospholipid which is known to regulate several cellular processes including cell motility, proliferation, survival and differentiation. Recent study showed the association of increased LPA and G-protein-coupled LPA1 receptor with the development of renal fibrosis^34^. Plasma LPA concentration was significantly increased in our CRF rats compared to the control group. LPA is produced by several enzymes including phospholipases A1/A2, lysophospholipase D/autotaxin, glycerol-phosphate acyltransferase, or monoacylglycerolkinase^35^. The expression and/or the activity of one of these enzymes might be increased in the rat kidney as an adaptive response to chronic kidney injury induced by adenine, which might be responsible for increased LPA level in the plasma from CRF group. CTGF is an important pro-fibrotic cytokine, signaling down-stream and in parallel with TGF-β. Earlier studies have shown that LPA can induce expression of the CTGF in cultured human fibroblasts^36^. Pradère *et al.* demonstrated that the pro-fibrotic activity of LPA in kidney could result from a direct action of LPA on kidney cells involving induction of CTGF^34^. Moreover, this induction was almost completely suppressed by co-treatment with the LPA receptor antagonist Ki16425^34^. This is consistent with the results of the present study in rats with CRF in which up-regulated CTGF was coupled with a marked increase in plasma LPA level and their parallel reductions with EA treatment (Fig. 3). Thus, EA treatment was able to ameliorate adenine-induced CRF by lowering CTGF expression. The biochemical mechanism of the anti-fibrotic effect of EA in the adenine-induced CRF rats might be mediated by inhibition of CTGF production via reduction of LPA1 level.

In summary, treatment with EA and BU extracts of rhubarb reversed the rise in plasma TG(55:1), PE(44:6), PE(38:4) and the decline in plasma TG(53:0), LPC(15:0) and DG(38:9) to normal level in CRF rats. The decline in PE(38:4) was reversed by EA extract. These observations suggest that rhubarb extracts can ameliorate some but not all adenine-induced CRF-associated abnormalities of glycerophospholipid metabolism.

L-carnitine is synthesized from the essential amino acids lysine and methionine. It can be transacylated to acyl-CoA. This acyl-CoA’s can then enter the β-oxidation pathway to form ATP (Fig. 10C). Carnitine deficiency has been shown to amplify the severity of kidney disease^37^. Patients with kidney disease frequently develop carnitine deficiency, especially those on dialysis. In this context urinary losses of carnitine can lead to carnitine deficiency in patients with renal tubular disorders^38^. In addition low serum and tissue-free carnitine concentrations and/or altered carnitine metabolism have been reported hemodialysis patients^39^,^40^. Plasma L-carnitine level was significantly reduced in our CRF rats, which is consistent with the reported findings in patients with kidney disease. The kidney plays an important role in the L-carnitine homeostasis. In healthy individuals, carnitine is freely filtered in the glomeruli and free carnitine is almost completely re-absorbed by proximal tubules. However, by limiting tubular reabsorption, renal tubular damage and dysfunction lead to urinary losses and depletion of carnitine in patients and animals with nephropathy^41^. This phenomenon may account for significant reduction of L-carnitine in our rats with adenine-induced tubulointerstitial nephropathy. In addition in patients with end-stage kidney disease removal of L-carnitine by dialysis procedure can lead to carnitine depletion. By limiting fatty acid oxidation and mitochondrial ATP generation, the associated L-carnitine deficiency can contribute to the prevailing weakness, fatigue, and impaired exercise capacity which are common features of advanced CRF^42^,^43^. L-carnitine has been also shown to attenuate the development of kidney fibrosis in rats with L-NAME induced hypertension by up-regulating PPAR-γ^44^ Attenuation of the renal fibrosis by L-carnitine in these animals was accompanied by significant increase in PPAR-γ expression, significant reduction of TGF-β1 and oxidative stress and inflammation markers. Furthermore, the anti-fibrotic effect of L-carnitine could be blocked by PPAR-γ inhibition. Taken together these findings illustrate the ability of L-carnitine in attenuating the development of fibrosis via PPAR-γ mediated down-regulation of TGF-β1, CTGF and α-SMA. Severe interstitial fibrosis and upregulation of kidney tissue TGF-β1, CTGF and α-SMA in rats with adenine-induced CRF was accompanied by marked reduction in plasma L-carnitine concentration. Given the severe renal tubulointerstitial injury in adenine-induced CRF model, the observed L-carnitine deficiency must be largely due to impaired tubular reabsorption and heavy urinary losses of L-carnitine. This leads to a vicious circle in which renal tubular damage causes L-carnitine deficiency and L-carnitine deficiency promotes renal interstitial fibrosis. Treatment with EA extract prevented the decline in plasma L-carnitine and L-acetylcarnitine and reduced expression of TGF-β1, CTGF and α-SMA in CRF rats. These observations suggest that the salutary effects of EA extract on renal interstitial fibrosis and tubular injury may be in part mediated by restoration of L-carnitine level.

Fatty acids are one of the simplest and the most important lipid classes. Fatty acids are precursors of various bioactive lipid molecules. Decreased plasma palmitic acid and ricinoleic acid were observed in our CRF rats. Palmitic acid which is one of the most common saturated fatty acids found in animals has been shown to induce endoplasmic reticulum stress and cause apoptotic and necrotic cell death in the renal proximal tubular cell line^45^. Our previous study demonstrated significant reduction of plasma palmitic acid in rats with adenine-induced CRF^46^. Administration of BU extract of rhubarb restored plasma palmitic acid to normal levels in our CRF rats. Likewise treatment with EA extract prevented the decline in ricinoleic acid in the CRF rats. Taken together, data obtained on L-carnitine, and fatty acid metabolism revealed the disturbance of energy metabolism in rats with adenine-induced CRF. Most but not all of these abnormalities were improved by administration of rhubarb extracts.

Our current study showed significantly elevated plasma hypotaurine level in rats with CRF, indicating disturbance of taurine metabolism. Hypotaurine is an intermediate in the biosynthesis of taurine and possesses anti-oxidant activity. It has been reported that urinary hypotaurine level is decreased but serum hypotaurine was increased in patients with CRF^47^. However neither EA nor BU or PE extracts could prevent the adenine-induced changes in plasma hypoxanthine levels. In addition, significantly increased plasma creatinine was observed in rats with CRF reflecting presence of renal insufficiency. Increased plasma creatinine was attenuated by treatment with EA extract pointing to improvement of renal function, which is consistent with result of clinical chemistry.

## Conclusion

In this study, we employed plasma lipidomics and metabolomics to investigate the pathophysiology of adenine-induced CRF in rats and its response treatment with different rhubarb extracts. The CRF group exhibited extensive renal tubulointerstitial injury and fibrosis, upregulation of the fibrotic proteins, marked reduction of renal function, and extensive changes of the plasma lipidomic and metabolomic profiles. Oral administration of PE, EA and BU extracts of rhubarb attenuated severity of renal lesions, improved renal function, reduced up-regulation of the fibrotic proteins, and partially reversed abnormalities of plasma lipidome and metabolome.

## Materials and Methods

### Chemicals and reagents

Creatinine and adenine were obtained from the National Institutes for Food and Drug Control and Sigma Chemical Co., Ltd. All the antibodies were purchased from Santa Cruz Biotechnology or Abcam Company. LC-grade methanol and acetonitrile were purchased from the Baker Company. Ultra high purity water was prepared using a Milli-Q water purification system. Other chemicals were of analytical grade and their purity was above 99.5%.

### Preparation of rhubarb extracts

Rhubarb was ground to powder and the powder was sieved by 20 meshes. Rhubarb powder (2 kg) was weighed and extracted with 15 L 95% ethanol for 0.5 h by ultrasonic method for three times. The resulting extract was evaporated to dryness under reduced pressure to yield a brown crude extract. Then the ethanol extract obtained was partitioned between water and three organic solvents with different polarities (BU > EA > PE), to yield three new fractions including PE, EA and BU extracts.

### Animals and sample collection

Male Sprague-Dawley rats (8 weeks old), weighting 200 ± 10 g were purchased from Fourth Military Medical University (Xi’an, China). The rats were randomized to divide into the following five groups (n = 8): control group, CRF group, CRF + PE group, CRF + EA group and CRF + BU group. CRF, CRF + PE, CRF + EA and CRF + BU groups were then given 200 mg/kg body weight of adenine dissolved in 1% (w/v) gum acacia solution by oral gavage once everyday continuously for three weeks^48^,^49^. Control group was similarly provided an equal volume of gum acacia solution. During the adenine gastric gavage periods, after 3 h, CRF + PE, CRF + EA and CRF + BU groups were administered PE extract (800 mg/kg), EA extract (200 mg/kg) and BU extract (600 mg/kg) by gastric irrigation respectively during six weeks study periods. The same amount of rhubarb powder was used to yield the PE (800 mg/kg), EA (200 mg/kg) and BU (600 mg/kg) extracts. The administrated dosage of PE, EA and BU/kg body weight in rats was 20 times the dosage of rhubarb recommended for humans by the Chinese Pharmacopeia All groups were only administered by oral gavage with the 1% (w/v) gum acacia solution. All the rats were anesthetized with 10% urethane, and blood samples were obtained by carotid artery cannula at week 6. Blood was centrifuged at 3000 rpm for 10 min and the supernatant was collected and stored at −80 °C before analysis. Kidney was harvested and immediately washed with physiological saline and stored at −80 °C for the following histological study. This study was approved by the Ethical Committee of Northwest University and studies were performed in accordance with the Guide for the Care and Use of Laboratory Animals defined by Ethical Committee of Northwest University.

### Biochemical determination

Plasma clinical biochemistry was analyzed as described in detail previously^46^ biochemical parameters were measured using an Olympus AU640 automatic analyzer. The measurements for each of the samples for biochemical parameters were replicated 3 times.

### PAS staining and Masson’s trichrome staining

PAS staining and Masson’s trichrome staining were performed as described in detail previously^50^,^51^. Cross-sections of the kidneys were fixed in 10% buffered formaldehyde solution and embedded in paraffin. The sections (5 μm) were obtained and used for histological and immunohistochemical analyses. The sections were stained with PAS method. The slides were examined under light microscopy by a pathologist in a blinded manner. Histological findings were graded according to the modified Banff classification criteria in 20 randomly selected non-overlapping fields per PAS staining.

For renal fibrosis, Masson’s trichrome staining was used to evaluate the extent of renal fibrosis according to the standard Masson’s trichrome protocol. Briefly, kidney tissue sections were successively immersed into Weigert’s iron hematoxylin, Biebrich scarlet-acid fuchsin, phosphomolybdic-phosphotungstic acid, and aniline blue. To quantify the renal fibrosis, the blue pixel contents of the images were photographed with the same microscope and magnification times. Ten different views in each group were selected to detect the values of the integral optical density and the total area and the expression intensity was calculated as the percentage of the integral optical density to the total area which was performed by Image-Pro Plus 6.0 (Media Cybernetics, Inc.). The measurements for each of the samples for PAS staining and Masson’s trichrome staining were replicated 3 times.

### Immunohistochemical staining

Immunohistochemical staining of ED-1 and α-SMA was performed as described in detail previously^52^,^53^. Briefly, kidney tissue sections were deparaffinised in xylene, hydrated using graded ethanol, and rinsed with tap water and distilled water. Then the endogenous peroxide activity was blocked using 0.3% hydrogen peroxide in methanol for 30 min. For antigen retrieval, the kidney tissue sections were incubated with 10 μmol/L citrate buffer solution (pH: 6.0) and boiled for 10-15 min. Subsequently, the kidney tissue sections were blocked with 10% normal goat serum for 1 h at room temperature and then incubated overnight at 4 °C with rabbit α-smooth muscle actin antibody (Abcam, ab5694, 1:200, Cambridge, UK) and a rat ED1 antibody (Abcam, ab31630, 1:400, Cambridge, UK). After washing with PBS, the secondary antibody (Abcam, ab7010, 1:500, Cambridge, UK) was added and the sections were incubated at 37 °C for 1 h. Finally, the kidney tissue sections were exposed to diaminobenzidine peroxidase substrate for 5 min and counterstained with hematoxylin and eosin. Image analysis was done by using Image-Pro Plus 6.0 software. The measurements for each of the samples for immunohistochemical staining were replicated 3 times.

### Western blot analysis

Western blot analysis including α-SMA, collagen I, FN, PAI-1, TGF-β1 and CTGF proteins were performed as described in detail previously^54^. Immunoblotting was performed using standard protocols with the following primary antibodies: rabbit collagen I antibody (Abcam, ab34710, 1:100, Cambridge, UK), rabbit FN antibody (Abcam, ab2413, 1:100, Cambridge, UK), mouse TGFβ-1 antibody (Abcam, ab64715, 1:400, Cambridge, UK), rabbit PAI antibody (Abcam, ab66705, 1:200, Cambridge, UK), rabbit CTGF antibody (Abcam, ab6992, 1:100, Cambridge, UK). A horseradish peroxidase-conjugated secondary antibody (1:1000, Pierce) was used to label the protein band, and blots were developed using enhanced chemiluminescence reagents by following a procedure provided by the manufacturer (Amersham Pharmacia Biotech, USA). The measurements for each of the samples for Western blot analysis were replicated 3 times.

### Sample preparation and UPLC-MS analysis

Plasma for metabonomics was prepared as described previously^46^. Plasma for lipidomics was prepared as followed: lipids extraction was performed in Ostro 96-well plate using a single-step in-well extraction. 100 μL of plasma was loaded into each well of a 2 mL Ostro sample preparation plate fitted onto a vacuum manifold. 300 μL of elution solvent (1:1, chloroform/methanol) was added to each well and mixed thoroughly by aspirating the mixture 10x using a micropipette. A vacuum of approximately 15” Hg was applied to the plate until the solvent was completely drained. This step was repeated with another 300 μL of chloroform and methanol with the total fraction three times reaching a total fraction volume of approximately 900 μL. The eluate fraction was dried under nitrogen, reconstituted with 200 μL 1:1 (v/v) chloroform/methanol, and then injected into the UPLC-MS system. Renal tissue for metabonomics was prepared as described previously^49^.

Lipidomics and Metabolomics were performed on a Waters Acquity^TM^ Ultra Performance LC system equipped with a Waters Xevo^TM^ G2-S QTof MS. Chromatographic separation, mass spectrometry were described in detail in the Supplementary information.

### Data processing and statistical analysis

The precision and reproducibility were tested for assessment of the developed UPLC-MS method following the published method^49^. The raw data were analyzed using Markerlynx XS, this allowed deconvolution, alignment and data reduction to give a list of mass and retention time pairs with corresponding intensities for all the detected peaks from each data file in the dataset. The main parameters were set as follows: retention time range 1–8 min for metabolomic analysis, retention time range 1–10 min for lipidomic analysis, mass range 50–1000 amu for metabolomic analysis, mass range 100–1500 amu for lipidomic analysis, mass tolerance 0.01, minimum intensity 1%, mass window 0.05, retention time window 0.20, and noise elimination level 6. The resulting three-dimensional matrix including sample names (observations), arbitrary compound index (*t*_m/z) and peak areas (variables) were introduced into the SIMCA-P 13.0 for multivariate statistical analysis. The spectral data were conducted by PCA to visualize general clustering, trends or outliers among the observations. OPLS-DA and PLS-DA was utilized to validate the PCA model and identify the differential metabolites. R^2^ represents the explanation capacity of the model (R^2^X and R^2^Y represent the fraction of the variance of X matrix and Y matrix), while Q^2^ suggests the predictive accuracy of the model. The cumulative values of R^2^X, R^2^Y and Q^2^ close to 1 indicate an excellent model. Metabolite peaks were assigned by MS data, MS^E^ fragments, molecular weights and elemental compositions or interpreted with available biochemical databases, such as HMDB (http://www.hmdb.ca/), KEGG (http://www.kegg.com/) and Chemspider (http://www.chemspider.com).

Fold changes from each group/control group or each group/CRF group and ROC curve was performed by Metaboanalysis 3.0. Student’s t test and Mann-Whitney test were used to calculate the statistical significance between two groups by SPSS 19.0. VIP was used to rank the contribution of each variable based on the PLS-DA model, and those variables with VIP > 1.0 are considered relevant for group discrimination^55^. The p value of both Student’s t test and Mann-Whitney test was set to 0.05 for this study. Based on the Hochberg-Benjamini method, the resultant p values from Student’s t test were further adjusted by a FDR.

## Additional Information

**How to cite this article**: Zhang, Z.-H. *et al.* An integrated lipidomics and metabolomics reveal nephroprotective effect and biochemical mechanism of Rheum officinale in chronic renal failure. *Sci. Rep.* **6**, 22151; doi: 10.1038/srep22151 (2016).

## Supplementary Material

Supplementary Information

## Figures and Tables

**Table 1:**
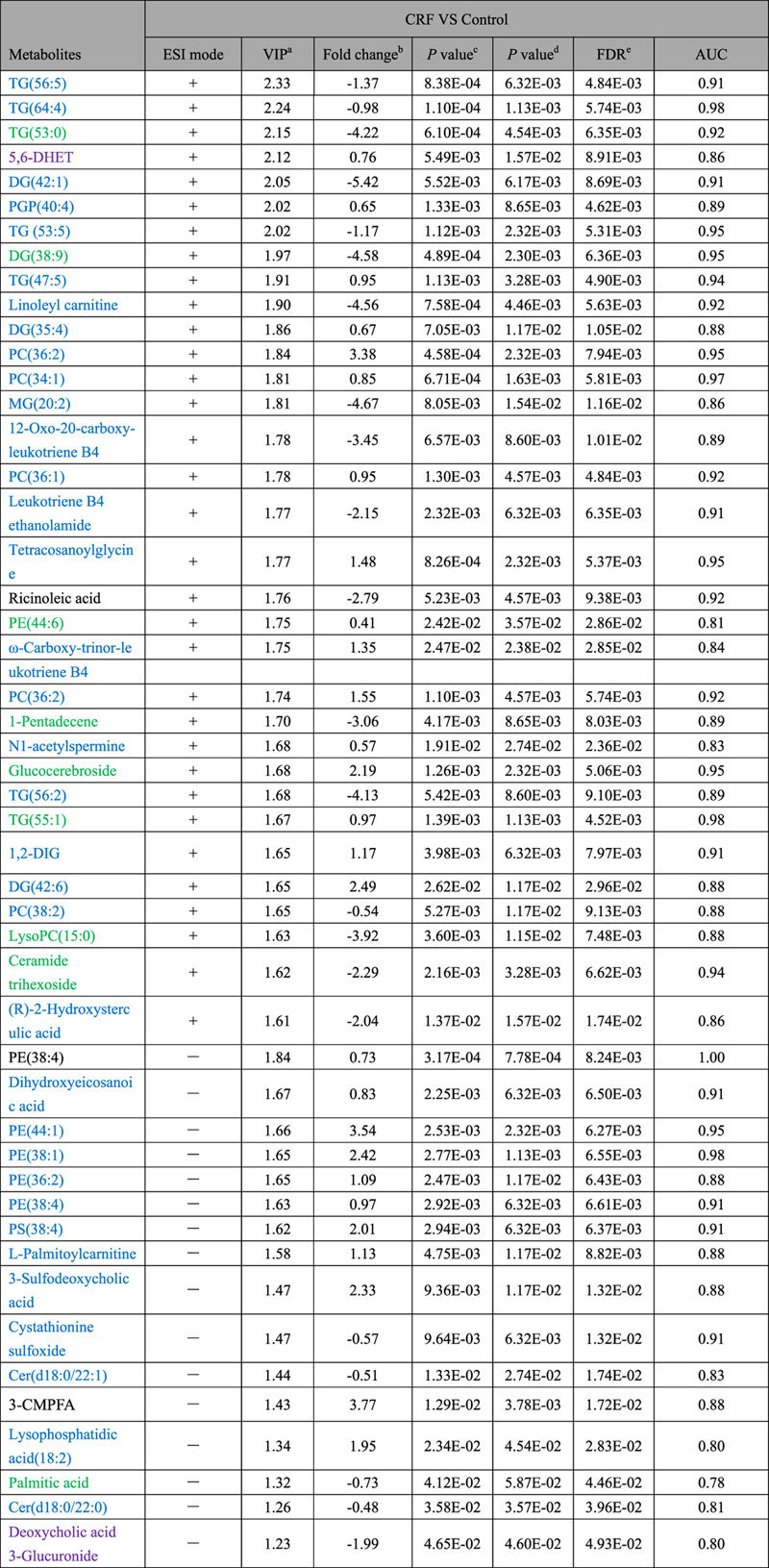
Differential plasma metabolites between control and CRF group based on lipidomic profile in positive and negative ion modes.

^a^VIP was obtained from the PLS-DA model.

^b^Fold change was calculated as a binary logarithm of the average mass response (peak area) ratio between CRF group vs control group, where a positive value means that the average mass response of the metabolite in CRF group is larger than that in the control group.

^c^The *p* value was calculated from Student’s *t* test.

^d^The *p* value was calculated from nonparametric test Mann−Whitney U test.

^e^All the metabolites were discriminant (Student’s t test *p* < 0.05), with an FDR of 5%. Metabolites with marked green indicate that their concentration were reversed to normal level by PE, EA or BU treatments; metabolites with marked purple indicate that their concentration were reversed to the level higher than normal level by PE, EA or BU treatments; metabolites with marked black indicate that their concentration were reversed by PE, EA or BU treatments; metabolites with marked blue indicate that their concentration can’t be reversed by PE, EA or BU treatments. 1,2-DIG: 1,2-Di-(9Z,12Z,15Z-octadecatrienoyl)-3-(galactosyl-alpha-1-6- galactosyl-beta-1)-glycerol.

**Table 2:**
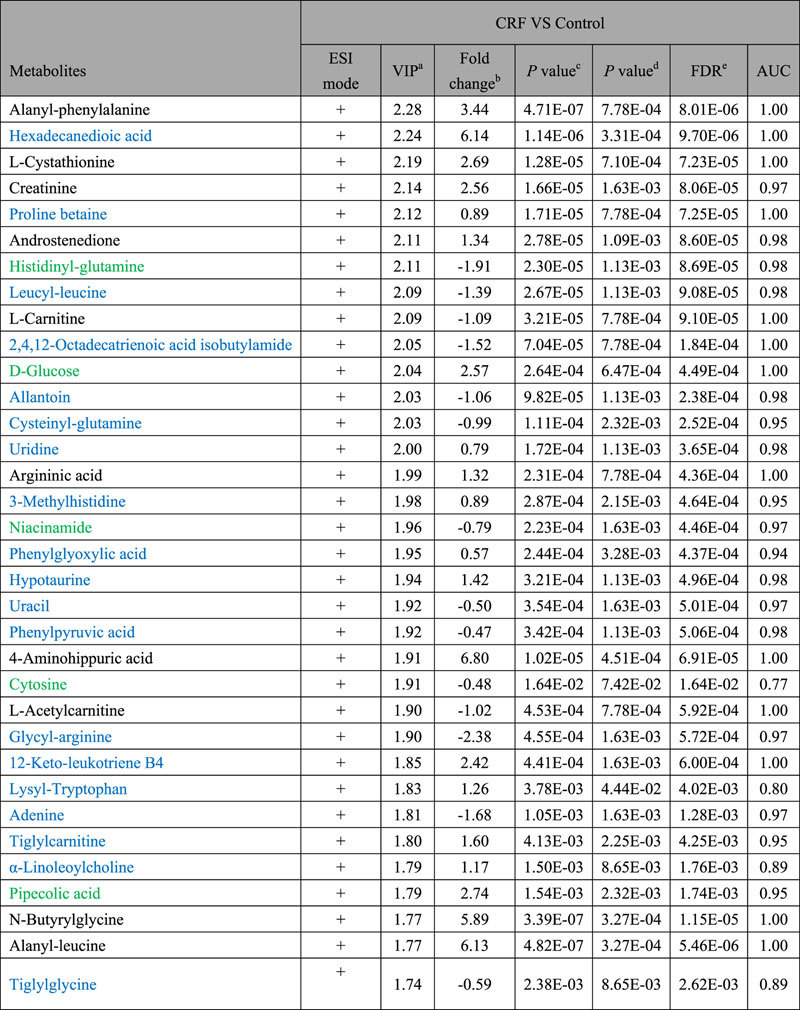
Differential plasma metabolites between control and CRF group based on metabolomic profile in positive ion mode.

^a^VIP was obtained from the PLS-DA model.

^b^Fold change was calculated as a binary logarithm of the average mass response (peak area) ratio between CRF group vs control group, where a positive value means that the average mass response of the metabolite in CRF group is larger than that in the control group.

^c^The *p* value was calculated from Student’s t test.

^d^The *p* value was calculated from nonparametric test Mann−Whitney U test.

^e^All the metabolites were discriminant (Student’s t test *p* < 0.05), with an FDR of 5%. Metabolites with marked green indicate that their concentration were reversed to normal level by PE, EA or BU treatments; metabolites with marked purple indicate that their concentration were reversed to the level higher than normal level by PE, EA or BU treatments; metabolites with marked black indicate that their concentration were reversed by PE, EA or BU treatments; metabolites with marked blue indicate that their concentration can’t be reversed by PE, EA or BU treatments.
