# Supplementary material for: An integrated lipidomics and metabolomics reveal nephroprotective effect and biochemical mechanism of Rheum officinale in chronic renal failure
Source: Sci Rep. 2016 Feb 23;6:22151. doi: 10.1038/srep22151 (PMC4763304; doi:10.1038/srep22151)
Supplement: Supplementary Information [file srep22151-s1.doc]

*Supporting Information*

An integrated lipidomics and metabolomics reveal nephroprotective effect and biochemical mechanism of Rheum officinale in chronic renal failure

Zhi-Hao Zhang ^3^, Nosratola D Vaziri ^2^, Feng Wei ^4^, Xian-Long Cheng ^4^, Xu Bai ^5^, Ying-Yong Zhao ^*,1,2^

^1^ Key Laboratory of Resource Biology and Biotechnology in Western China, Ministry of Education, the College of Life Sciences, Northwest University, No. 229 Taibai North Road, Xi’an, Shaanxi 710069, China

^2^ Division of Nephrology and Hypertension, School of Medicine, University of California, Irvine, MedSci 1, C352, UCI Campus, Irvine, California, 92697, USA

^3^National Center for Natural Products Research, Department of BioMolecular Sciences, School of Pharmacy, University of Mississippi, Oxford, Mississippi, 38677, USA

^4^ National Institutes for Food and Drug Control, State Food and Drug Administration, No. 2 Tiantan Xili, Beijing, 100050, China

^5^ Solution Centre, Waters Technologies Ltd., No. 1000 Jinhai Road, Shanghai 201203, China

Corresponding author:

Professor Ying-Yong Zhao, PhD, MD, Tel: +86 29 88304569; Fax: +86 29 88304368; E-mail: zyy@nwu.edu.cn; zhaoyybr@163.com (Y.Y. Zhao)

**Chromatographic separation of metabolomics analysis**

Each sample was injected onto a reverse-phase 100 × 2.1 mm, HSS 1.7 µm C_18_ column using an ACQUITY UPLC system (Waters Corporation, USA). The gradient mobile phase comprised of water containing 0.1% formic acid solution (A) and acetonitrile (B). Each sample was resolved for 9 min at a flow rate of 0.45 mL/min. The mobile phase consisted of 0.1% formic acid water (A) and acetonitrile (B). The optimized UPLC elution conditions were: 0.0–7.0 min, 99.0–1.0% A; 7.0–8.0 min, 1.0% A and 8.0–9.0 min, 1.0–99.0% A. The autosampler was maintained at 4 °C. Every 5 µL sample solution was injected for each run.

**Mass spectrometry of metabolomics analysis**

The column eluent was introduced directly into the mass spectrometer by electrospray. Mass spectrometry was performed on a quadrupole-time-of-flight mass spectrometer operating in either negative or positive electrospray ionization mode with a capillary voltage of 3.0 kV and a sampling cone voltage of 35 V. The desolvation gas flow was 600 L/h and the temperature was set to 350 °C. The cone gas flow was 50 L/h, and the source temperature was 100 °C. The mass spectrometry was operated in W optics mode with 12,000 resolution using dynamic range extension. Data were acquired in continuum mode from 50 to 1000 m/z mass range for TOF-MS scanning, in duplicates (technical replicates) for each sample in positive and negative ionization mode and checked for chromatographic reproducibility. Leucine–enkephalin was used as the lockmass at a concentration of 300 ng/mL and flow rate of 5 µL/min. Data were collected in continuum mode, the lockspray frequency was set at 10 s, and data were averaged over 10 scans. All the acquisition and analysis of data were controlled by Waters Unifi software.

**Chromatographic separation of lipidomics analysis**

The UPLC analysis was performed in the above-mentioned chromatographic separation of metabolomic analysis. A gradient of 10 mM ammonium formate in 2-propanol/acetonitrile (90/10) in 0.1% formic acid (A) and 10 mM ammonium formate in ACN/H_2_O (60/40) in 0.1% formic acid (B) was used as follows: a linear gradient of 0–10 min, 40.0–99.0% A and 10.0–12.0 min, 99.0–40.0% A. The flow rate was 0.5 ml/min. The temperatures of autosampler and chromatographic column were maintained at 4 °C and 55 °C, respectively. Every 5 µL sample solution was injected for each run.

**Mass spectrometry of lipidomics analysis**

For lipidomics analysis, mass spectrometry was performed on a Xevo^TM^ G2 QTof. The scan range was from 100 to 1500 m/z. For both positive and negative electrospray modes, the capillary and cone voltage were set at 3.0 kV and 60 V, respectively. The desolvation gas was set to 900 L/h at a temperature of 500 °C; the cone gas was set to 50 L/h and the source temperature was set to 120 °C. An MS^E^ experiment was performed as follows: function 1, 10 V collision energy; function 2, collision energy ramp of 20–65 V. Data were collected in continuum mode, the lockspray frequency was set at 10 s, and data were averaged over 10 scans. All the acquisition and analysis of data were controlled by Waters Unifi software.

Figure S1


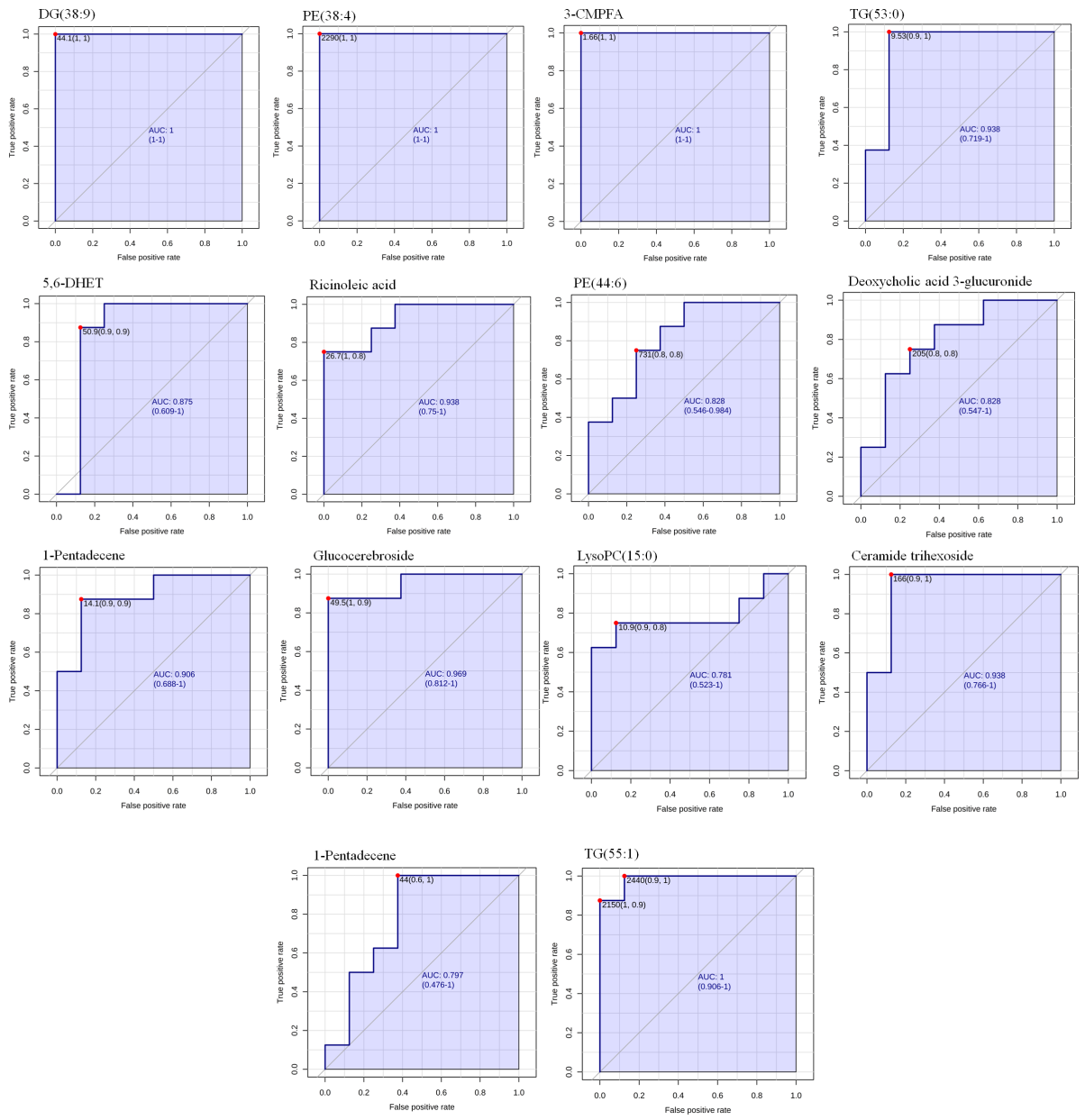


Figure S1. PLS-DA-based receive operating characteristic curves of the biomarkers from lipidomic samples for evaluation of nephroprotective effects of EA, BU and PE treatments. The associated area under the curve (AUC), 95% confidence interval (95%CI), sensitivities and specificities were indicated.

Figure S2


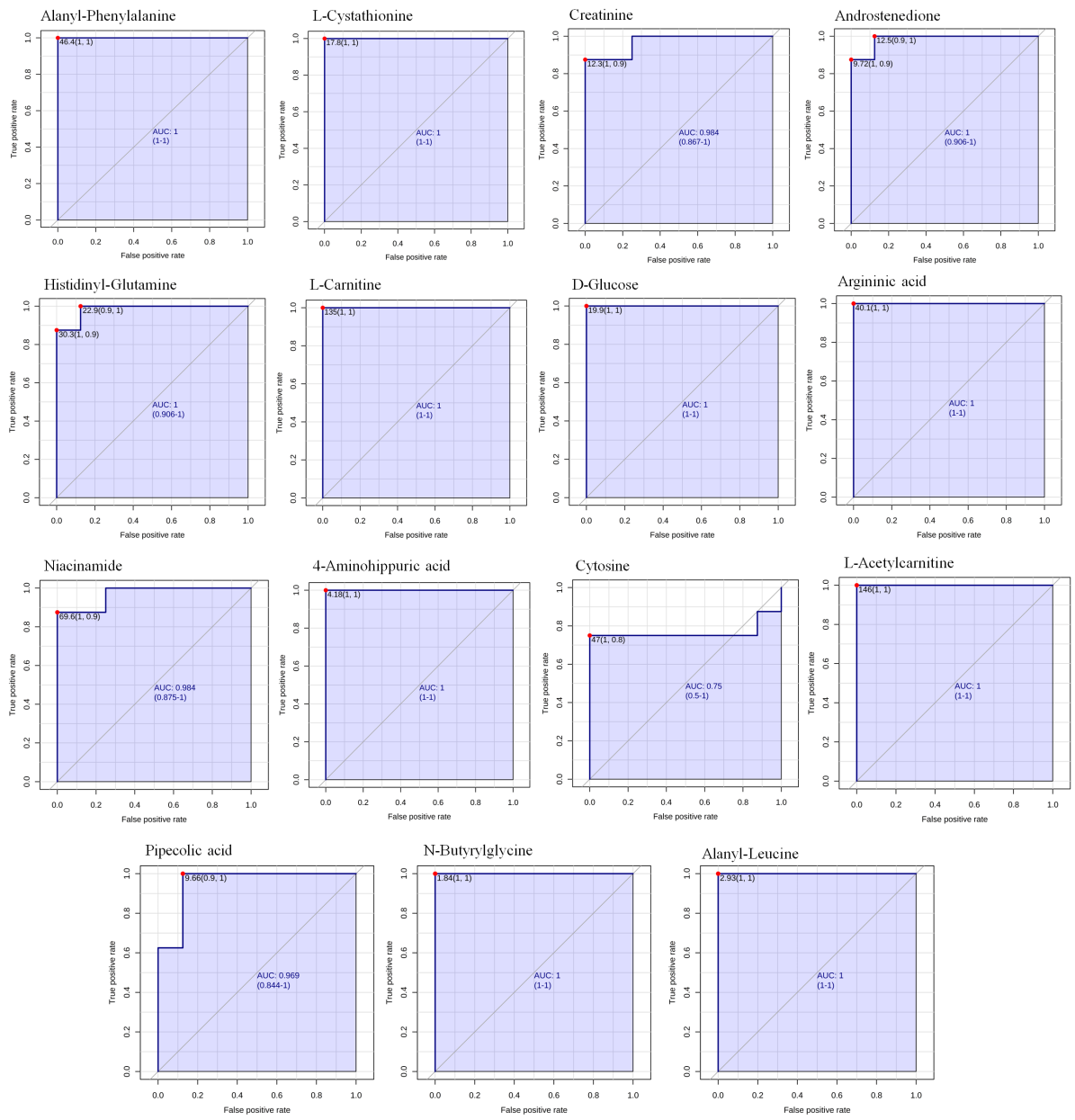


Figure S2. PLS-DA-based receive operating characteristic curves of the biomarkers from metabolomic samples for evaluation of nephroprotective effects of EA, BU and PE treatments. The associated area under the curve (AUC), 95% confidence interval (95%CI), sensitivities and specificities were indicated.

Figure S3

A.

B

Figure S3. The representative BPI chromatograms (ESI^+^) of lipidomic samples from control group (A) and CRF group (B).

Figure S4

A

B

Figure S4. The representative BPI chromatograms (ESI^-^) of lipidomic samples from control group (A) and CRF group (B).

Table S1. Comparison of the concentration of these 49 metabolites among all groups including CRF animals treated with different extracts based on lipidomic profiling in positive and negative ion mode

| Primary ID | PE VS Control | |  | PE VS CRF | |  | EA VS Control | |  | EA VS CRF | |  | BU VS Control | |  | BU VS CRF | |
| --- | --- | --- | --- | --- | --- | --- | --- | --- | --- | --- | --- | --- | --- | --- | --- | --- | --- |
|  | Fold change^a^ | *P* value^b^ |  | Fold change^a^ | *P* value^b^ |  | Fold change^a^ | *P* value^b^ |  | Fold change^a^ | *P* value^b^ |  | Fold change^a^ | *P* value^b^ |  | Fold change^a^ | *P* value^b^ |
| TG(16:0/o-18:0/22:5) | -1.36 | 8.71E-04 |  | -0.05 | 9.14E-01 |  | -1.15 | 3.84E-04 |  | 0.22 | 7.72E-01 |  | -3.00 | 2.34E-03 |  | -1.63 | 7.84E-01 |
| TG(18:2/24:0/22:2) | -1.21 | 2.28E-05 |  | -0.50 | 1.25E-01 |  | -1.06 | 1.74E-04 |  | -0.08 | 9.29E-01 |  | -1.47 | 1.83E-05 |  | -0.49 | 1.60E-01 |
| TG(15:0/22:0/16:0) | -0.79 | 8.67E-03 |  | 1.50 | 8.19E-04 |  | -0.76 | 2.52E-01 |  | 1.84 | 9.41E-07 |  | -2.12 | 2.10E-03 |  | 0.48 | 5.99E-04 |
| 5,6-DHET | 0.56 | 7.33E-03 |  | -0.07 | 5.52E-02 |  | 0.73 | 4.36E-04 |  | -0.03 | 6.78E-01 |  | -1.54 | 3.94E-03 |  | -2.30 | 2.47E-04 |
| DG(18:0/0:0/24:1n9) | -1.98 | 5.74E-03 |  | -0.29 | 4.71E-01 |  | -1.90 | 7.85E-03 |  | -0.49 | 6.55E-01 |  | -2.07 | 1.13E-02 |  | -0.66 | 8.48E-01 |
| PGP(18:0/22:4) | 0.55 | 2.21E-02 |  | -0.03 | 7.03E-01 |  | 0.26 | 2.49E-01 |  | -0.40 | 2.18E-01 |  | 0.28 | 2.60E-01 |  | -0.37 | 1.63E-01 |
| TG(15:0/20:5/o-18:0) | -1.53 | 2.05E-04 |  | -0.74 | 2.79E-01 |  | -0.63 | 7.15E-03 |  | 0.54 | 1.40E-01 |  | -2.02 | 9.16E-06 |  | -0.85 | 3.94E-02 |
| DG(18:4/20:5/0:0) | -2.46 | 6.96E-04 |  | -0.39 | 7.82E-01 |  | -0.64 | 3.02E-01 |  | 1.93 | 3.78E-04 |  | -1.82 | 5.61E-04 |  | 0.75 | 5.94E-02 |
| TG(15:0/14:1/18:4) | 0.29 | 2.21E-02 |  | -0.22 | 9.71E-01 |  | 1.20 | 2.55E-04 |  | 0.25 | 1.51E-01 |  | 1.18 | 4.60E-04 |  | 0.23 | 1.90E-01 |
| Linoleyl carnitine | -1.61 | 1.54E-03 |  | -0.80 | 1.19E-01 |  | -1.49 | 1.90E-03 |  | -0.20 | 6.83E-01 |  | -4.57 | 4.20E-05 |  | -3.28 | 7.63E-09 |
| DG(20:4/15:0/0:0) | 0.50 | 3.50E-02 |  | -0.18 | 3.31E-01 |  | 0.56 | 2.69E-02 |  | -0.11 | 6.28E-01 |  | 0.92 | 3.34E-03 |  | 0.25 | 2.46E-01 |
| PC(20:1/16:1) | 1.91 | 1.00E-04 |  | 0.00 | 8.82E-01 |  | 3.01 | 2.33E-03 |  | 0.02 | 5.69E-01 |  | 3.96 | 2.00E-05 |  | 0.53 | 1.18E-01 |
| PC(18:1)/16:0) | 0.67 | 2.02E-03 |  | 0.00 | 9.05E-01 |  | 0.96 | 2.83E-04 |  | 0.11 | 5.53E-01 |  | 0.77 | 7.00E-04 |  | -0.08 | 6.15E-01 |
| MG(20:2/0:0/0:0) | -1.24 | 5.51E-03 |  | -0.39 | 2.96E-01 |  | -0.63 | 1.06E-01 |  | 0.47 | 3.30E-01 |  | -5.60 | 8.24E-05 |  | -4.51 | 4.79E-04 |
| 12-Oxo-20-carboxy-leukotriene B4 | -0.70 | 7.13E-02 |  | 2.49 | 1.76E-01 |  | -0.93 | 1.42E-01 |  | 2.52 | 1.34E-01 |  | -2.65 | 8.72E-03 |  | 0.91 | 8.33E-01 |
| PC(o-16:1/20:0) | 0.43 | 2.59E-02 |  | -0.32 | 6.82E-01 |  | 0.78 | 8.50E-03 |  | -0.17 | 5.03E-01 |  | 0.69 | 7.25E-03 |  | -0.26 | 1.32E-01 |
| Leukotriene B4 ethanolamide | -0.87 | 7.22E-03 |  | 1.30 | 2.77E-01 |  | -0.74 | 3.64E-02 |  | 1.41 | 1.46E-01 |  | -1.86 | 1.65E-04 |  | 0.34 | 1.99E-01 |
| Tetracosanoylglycine | 1.69 | 8.37E-04 |  | 0.23 | 4.05E-01 |  | 1.27 | 8.46E-03 |  | -0.21 | 9.23E-01 |  | 0.53 | 1.86E-01 |  | -0.94 | 9.85E-02 |
| Ricinoleic acid | -2.46 | 1.10E-02 |  | -0.60 | 3.65E-01 |  | -1.85 | 1.35E-02 |  | 0.94 | 4.12E-02 |  | -3.61 | 3.75E-03 |  | -0.82 | 5.42E-03 |
| PE(22:1/22:5) | 0.39 | 1.19E-01 |  | -0.11 | 6.94E-01 |  | -0.02 | 9.99E-01 |  | -0.43 | 3.76E-02 |  | -0.24 | 1.97E-01 |  | -0.65 | 3.82E-03 |
| Omega-Carboxy-trinor-leukotriene B4 | 0.88 | 7.81E-02 |  | -0.36 | 7.58E-01 |  | 1.97 | 1.96E-02 |  | 0.98 | 9.32E-01 |  | 3.49 | 1.60E-03 |  | 2.14 | 4.28E-02 |
| PC(14:1/22:1) | 1.01 | 2.64E-03 |  | -0.16 | 8.80E-01 |  | 1.89 | 2.36E-04 |  | 0.34 | 1.92E-01 |  | 2.07 | 3.06E-06 |  | 0.52 | 3.09E-02 |
| 1-Pentadecene | -0.69 | 3.08E-01 |  | 1.63 | 7.43E-02 |  | -0.73 | 2.50E-01 |  | 2.33 | 1.19E-01 |  | -0.98 | 8.63E-01 |  | 2.17 | 1.20E-02 |
| N1-Acetylspermine | 0.19 | 1.12E-01 |  | -0.22 | 3.43E-01 |  | 0.59 | 8.95E-03 |  | 0.02 | 9.44E-01 |  | 0.36 | 6.31E-02 |  | -0.21 | 2.31E-01 |
| Glucosylceramide (d18:1/24:1) | 0.99 | 1.17E-01 |  | -1.26 | 2.89E-02 |  | 1.57 | 4.20E-02 |  | -0.61 | 9.51E-01 |  | 2.19 | 3.09E-03 |  | 0.01 | 4.44E-01 |
| TG(18:1/18:0/20:1) | -1.69 | 3.02E-02 |  | 1.44 | 4.96E-01 |  | -1.93 | 2.22E-02 |  | 2.18 | 4.44E-01 |  | -2.19 | 2.24E-01 |  | 2.08 | 2.30E-01 |
| TG(15:0/18:1/22:0) | 0.53 | 2.04E-04 |  | -0.29 | 4.57E-01 |  | -0.28 | 6.23E-01 |  | -1.25 | 2.35E-02 |  | 0.14 | 3.33E-01 |  | -0.83 | 2.97E-02 |
| 1,2-Di-(9Z,12Z,15Z-octadecatrienoyl)-3-(Galactosyl-alpha-1-6-Galactosyl-beta-1)-glycerol | 0.38 | 1.77E-02 |  | -0.70 | 8.27E-01 |  | 1.16 | 2.13E-03 |  | -0.01 | 8.58E-01 |  | 1.21 | 4.20E-04 |  | 0.04 | 9.36E-01 |
| DG(20:2n6/0:0/22:2n6) | 0.72 | 4.78E-02 |  | -1.25 | 5.95E-01 |  | 2.14 | 6.48E-03 |  | 0.02 | 7.62E-01 |  | 2.34 | 1.23E-02 |  | -0.20 | 3.66E-01 |
| PC(P-18:1/20:1) | -0.41 | 1.50E-01 |  | 0.13 | 5.32E-01 |  | -0.67 | 1.70E-04 |  | -0.13 | 3.80E-01 |  | -0.95 | 3.18E-05 |  | -0.41 | 5.80E-02 |
| LysoPC(15:0) | -0.91 | 1.08E-02 |  | -0.14 | 5.52E-01 |  | -0.46 | 3.81E-01 |  | 0.51 | 9.20E-02 |  | -0.12 | 8.07E-01 |  | 0.84 | 3.22E-02 |
| Trihexosylceramide (d18:1/12:0) | -2.26 | 2.65E-04 |  | -0.89 | 2.21E-01 |  | -0.11 | 7.50E-01 |  | 2.18 | 2.40E-03 |  | 0.47 | 9.06E-02 |  | 2.81 | 2.45E-03 |
| (R)-2-Hydroxysterculic acid | -1.32 | 6.25E-02 |  | 0.01 | 6.38E-01 |  | -0.74 | 2.50E-01 |  | 1.30 | 2.30E-01 |  | -0.82 | 6.76E-02 |  | 1.27 | 5.83E-01 |
| PE(18:1/20:3) | 0.35 | 8.14E-03 |  | -0.26 | 1.73E-01 |  | 0.37 | 7.63E-03 |  | -0.36 | 3.01E-02 |  | 0.56 | 2.54E-03 |  | -0.17 | 3.28E-01 |
| 10,20-Dihydroxyeicosanoic acid | 0.44 | 9.31E-02 |  | -0.44 | 1.80E-01 |  | 0.64 | 2.28E-02 |  | -0.18 | 5.70E-01 |  | 0.84 | 1.04E-03 |  | 0.01 | 9.92E-01 |
| PE(20:0/24:1) | 1.53 | 1.36E-05 |  | -0.26 | 2.35E-01 |  | 3.02 | 8.12E-03 |  | -0.52 | 5.47E-01 |  | 3.44 | 5.87E-03 |  | -0.10 | 8.23E-01 |
| PE(18:1/20:0) | 0.78 | 5.58E-03 |  | -1.00 | 3.00E-01 |  | 1.97 | 3.49E-03 |  | -0.44 | 3.35E-01 |  | 2.25 | 4.61E-05 |  | -0.17 | 4.30E-01 |
| PI(18:1/18:1) | 0.65 | 4.63E-02 |  | -0.39 | 1.12E-01 |  | 0.71 | 7.91E-02 |  | -0.39 | 3.28E-01 |  | 1.53 | 9.53E-04 |  | 0.44 | 9.52E-02 |
| PE(18:4/20:0) | 0.28 | 3.46E-02 |  | -0.53 | 3.31E-01 |  | 0.65 | 3.38E-03 |  | -0.32 | 1.67E-01 |  | 0.89 | 8.43E-04 |  | -0.09 | 6.13E-01 |
| PS(18:0/20:4) | -1.00 | 4.35E-02 |  | -1.21 | 3.76E-01 |  | 1.51 | 5.16E-02 |  | -0.49 | 1.93E-01 |  | 2.14 | 6.70E-05 |  | 0.13 | 7.23E-01 |
| L-Palmitoylcarnitine | 0.17 | 8.82E-02 |  | -0.76 | 3.37E-01 |  | 1.01 | 1.30E-02 |  | -0.11 | 7.57E-01 |  | 1.19 | 8.84E-04 |  | 0.07 | 9.08E-01 |
| 3-Sulfodeoxycholic acid | -0.54 | 2.96E-01 |  | -1.30 | 1.96E-01 |  | 2.55 | 1.17E-03 |  | 0.21 | 2.93E-01 |  | 2.64 | 4.32E-03 |  | 0.30 | 2.01E-01 |
| Cystathionine sulfoxide | -0.82 | 4.35E-04 |  | -0.38 | 1.83E-01 |  | -1.34 | 6.71E-04 |  | -0.77 | 1.87E-01 |  | -2.40 | 6.68E-03 |  | -1.83 | 2.63E-01 |
| Cer(d18:0/22:1) | -0.37 | 1.67E-01 |  | 0.32 | 9.73E-02 |  | -0.64 | 1.15E-02 |  | -0.13 | 7.65E-01 |  | -0.63 | 1.08E-02 |  | -0.12 | 8.06E-01 |
| 3-Carboxy-4-methyl-5-propyl-2-furanpropionic acid | 3.55 | 3.01E-11 |  | -1.02 | 8.49E-04 |  | 4.09 | 1.94E-19 |  | -0.98 | 6.52E-04 |  | 5.17 | 6.33E-04 |  | 0.09 | 8.10E-01 |
| Lysophosphatidic acid(18:2) | -0.23 | 3.61E-01 |  | -1.03 | 2.37E-01 |  | 3.22 | 1.69E-04 |  | 1.27 | 9.95E-02 |  | 3.70 | 4.95E-05 |  | 1.75 | 6.21E-03 |
| Palmitic acid | -0.25 | 2.54E-01 |  | 0.31 | 5.05E-01 |  | -0.06 | 9.52E-01 |  | 0.67 | 1.18E-01 |  | 0.03 | 8.43E-01 |  | 0.76 | 3.01E-04 |
| Cer(d18:0/22:0) | -0.40 | 1.89E-01 |  | 0.30 | 1.42E-01 |  | -0.60 | 4.64E-02 |  | -0.12 | 8.87E-01 |  | -0.47 | 1.54E-01 |  | 0.01 | 5.96E-01 |
| Deoxycholic acid 3-glucuronide | -2.26 | 8.98E-02 |  | -0.84 | 9.99E-01 |  | -0.66 | 8.31E-01 |  | 1.33 | 2.76E-01 |  | 1.28 | 1.47E-02 |  | 3.27 | 2.48E-03 |
| ^a^Fold change was calculated as a binary logarithm of the average mass response (peak area) ratio between each group vs control group or between each group vs CRF group, where a positive value means that the average mass response of the metabolite in each group is larger than that in the control group or CRF group. ^b^The *p* value was calculated from Student’s t test. | | | | | | | | | | | | | | | | | |

Table S2. Comparison of the concentration of these 34 metabolites among all groups including CRF animals treated with different extracts based on metabolomic profiling in positive ion mode

| Primary ID | PE VS Control | |  | PE VS CRF | |  | EA VS Control | |  | EA VS CRF | |  | BU VS Control | |  | BU VS CRF | |
| --- | --- | --- | --- | --- | --- | --- | --- | --- | --- | --- | --- | --- | --- | --- | --- | --- | --- |
|  | Fold change^a^ | *P* value^b^ |  | Fold change^a^ | *P* value^b^ |  | Fold change^a^ | *P* value^b^ |  | Fold change^a^ | *P* value^b^ |  | Fold change^a^ | *P* value^b^ |  | Fold change^a^ | *P* value^b^ |
| Alanyl-Phenylalanine | 2.94 | 1.66E-07 |  | -0.50 | 2.00E-02 |  | 2.38 | 6.61E-03 |  | -1.05 | 2.93E-02 |  | 3.28 | 2.89E-03 |  | -0.16 | 8.93E-01 |
| Hexadecanedioic acid | 5.65 | 6.68E-03 |  | -0.49 | 7.18E-01 |  | 5.67 | 6.43E-06 |  | -0.47 | 1.40E-01 |  | 6.18 | 1.97E-03 |  | 0.04 | 5.52E-01 |
| L-Cystathionine | 2.11 | 3.05E-05 |  | -0.59 | 5.51E-02 |  | 1.05 | 1.21E-02 |  | -1.64 | 7.92E-04 |  | 1.44 | 5.36E-03 |  | -1.25 | 4.60E-01 |
| Creatinine | 2.40 | 3.93E-04 |  | -0.17 | 5.24E-01 |  | 1.95 | 7.70E-03 |  | -0.61 | 5.40E-03 |  | 2.29 | 3.27E-03 |  | -0.28 | 5.41E-01 |
| Proline betaine | 0.83 | 1.66E-03 |  | -0.06 | 8.60E-01 |  | 0.77 | 5.32E-04 |  | -0.12 | 4.00E-01 |  | 1.04 | 2.19E-04 |  | 0.15 | 2.78E-01 |
| Androstenedione | 0.77 | 3.13E-02 |  | -0.57 | 4.31E-01 |  | 0.67 | 2.51E-02 |  | -0.67 | 2.33E-02 |  | 1.44 | 3.81E-03 |  | 0.10 | 5.42E-01 |
| Histidinyl-Glutamine | -0.44 | 1.81E-01 |  | 1.47 | 2.58E-03 |  | -0.04 | 8.78E-01 |  | 1.87 | 5.52E-04 |  | 0.23 | 3.09E-01 |  | 2.13 | 3.41E-04 |
| Leucyl-Leucine | -3.37 | 2.03E-08 |  | -1.98 | 8.08E-03 |  | -2.34 | 3.23E-08 |  | -0.95 | 2.44E-02 |  | -3.45 | 5.26E-09 |  | -2.06 | 2.80E-03 |
| L-Carnitine | -1.04 | 5.87E-05 |  | 0.04 | 7.08E-01 |  | -0.74 | 1.52E-03 |  | 0.35 | 4.18E-02 |  | -1.46 | 3.35E-06 |  | -0.37 | 7.74E-03 |
| 2,4,12-Octadecatrienoic acid isobutylamide | -3.47 | 2.02E-05 |  | -1.95 | 3.01E-01 |  | -2.16 | 1.00E-05 |  | -0.64 | 2.86E-01 |  | -4.50 | 9.10E-08 |  | -2.98 | 2.41E-03 |
| D-Glucose | 2.01 | 3.30E-06 |  | -0.57 | 3.33E-02 |  | -0.25 | 2.23E-01 |  | -2.82 | 1.45E-03 |  | 2.32 | 7.41E-03 |  | -0.25 | 9.50E-01 |
| Allantoin | -1.45 | 5.75E-06 |  | -0.38 | 4.46E-02 |  | -2.03 | 6.43E-07 |  | -0.97 | 3.14E-04 |  | -2.36 | 2.49E-07 |  | -1.30 | 3.04E-05 |
| Cysteinyl-Glutamine | -0.70 | 8.50E-03 |  | 0.29 | 1.15E-01 |  | -0.75 | 1.57E-03 |  | 0.24 | 1.35E-01 |  | -0.92 | 4.40E-04 |  | 0.07 | 6.23E-01 |
| Uridine | 0.78 | 6.32E-05 |  | -0.01 | 9.06E-01 |  | 0.72 | 3.52E-04 |  | -0.07 | 6.52E-01 |  | 0.94 | 2.95E-03 |  | 0.15 | 4.36E-01 |
| Argininic acid | 0.88 | 1.52E-02 |  | -0.43 | 2.03E-01 |  | 0.50 | 1.30E-02 |  | -0.82 | 3.66E-03 |  | 1.34 | 1.42E-04 |  | 0.02 | 9.00E-01 |
| 3-Methylhistidine | 0.83 | 1.64E-02 |  | -0.06 | 8.36E-01 |  | -0.83 | 4.99E-01 |  | -1.72 | 1.21E-01 |  | 0.01 | 1.10E-01 |  | -0.88 | 6.19E-01 |
| Niacinamide | -0.81 | 4.01E-03 |  | -0.02 | 6.53E-01 |  | -0.45 | 1.64E-02 |  | 0.34 | 6.17E-02 |  | -0.12 | 6.93E-01 |  | 0.67 | 6.85E-03 |
| Phenylglyoxylic acid | 0.70 | 4.30E-05 |  | 0.13 | 2.66E-01 |  | 0.59 | 7.73E-05 |  | 0.03 | 8.31E-01 |  | 0.50 | 9.93E-03 |  | -0.07 | 7.94E-01 |
| Hypotaurine | 1.57 | 1.11E-05 |  | 0.15 | 5.83E-01 |  | 1.31 | 3.03E-05 |  | -0.11 | 4.82E-01 |  | 1.30 | 1.15E-02 |  | -0.12 | 9.38E-01 |
| Uracil | -0.35 | 8.64E-04 |  | 0.14 | 2.87E-01 |  | -0.22 | 2.52E-01 |  | 0.27 | 1.30E-01 |  | -0.51 | 5.27E-04 |  | -0.02 | 9.59E-01 |
| Phenylpyruvic acid | -0.55 | 3.02E-04 |  | -0.08 | 6.21E-01 |  | -0.56 | 3.46E-05 |  | -0.09 | 4.27E-01 |  | -0.36 | 3.73E-03 |  | 0.12 | 3.53E-01 |
| 4-Aminohippuric acid | 5.37 | 4.76E-04 |  | -1.42 | 4.47E-02 |  | 3.54 | 4.03E-02 |  | -3.26 | 2.70E-02 |  | 5.65 | 1.22E-02 |  | -1.15 | 7.79E-01 |
| Cytosine | -0.16 | 2.76E-01 |  | 0.32 | 8.59E-03 |  | -0.43 | 7.41E-02 |  | 0.05 | 5.09E-01 |  | -0.52 | 3.04E-02 |  | -0.04 | 9.97E-01 |
| L-Acetylcarnitine | -0.78 | 2.80E-03 |  | 0.24 | 9.25E-02 |  | -0.74 | 2.44E-03 |  | 0.28 | 9.43E-03 |  | -0.89 | 1.26E-03 |  | 0.13 | 2.99E-01 |
| Glycyl-Arginine | -2.91 | 9.38E-05 |  | -0.53 | 2.76E-01 |  | -0.98 | 4.85E-03 |  | 1.40 | 6.83E-02 |  | -1.65 | 4.04E-04 |  | 0.73 | 8.26E-01 |
| 12-Keto-leukotriene B4 | 2.79 | 1.99E-06 |  | 0.37 | 3.78E-01 |  | 2.35 | 4.64E-06 |  | -0.08 | 6.13E-01 |  | 2.22 | 4.65E-04 |  | -0.20 | 5.58E-01 |
| Lysyl-Tryptophan | 1.79 | 1.16E-04 |  | 0.53 | 2.77E-01 |  | 1.04 | 3.04E-05 |  | -0.22 | 1.76E-01 |  | 0.69 | 3.21E-02 |  | -0.56 | 9.70E-02 |
| Adenine | -2.82 | 1.83E-05 |  | -1.14 | 1.80E-01 |  | -0.80 | 1.13E-04 |  | 0.89 | 5.28E-01 |  | -0.83 | 4.53E-02 |  | 0.85 | 3.11E-01 |
| Tiglylcarnitine | 1.01 | 2.54E-02 |  | -0.59 | 3.25E-01 |  | 1.40 | 2.97E-02 |  | -0.21 | 1.42E-01 |  | 1.85 | 5.33E-03 |  | 0.24 | 4.43E-01 |
| Alpha-Linoleoylcholine | 1.53 | 2.88E-05 |  | 0.36 | 8.48E-02 |  | 1.51 | 8.32E-05 |  | 0.34 | 1.15E-01 |  | 1.56 | 5.38E-05 |  | 0.39 | 7.33E-02 |
| Pipecolic acid | 2.10 | 4.58E-03 |  | -0.64 | 7.71E-02 |  | 0.94 | 1.56E-01 |  | -1.80 | 6.32E-04 |  | 1.33 | 2.98E-02 |  | -1.41 | 2.52E-01 |
| N-Butyrylglycine | 5.19 | 2.94E-06 |  | -0.70 | 1.44E-02 |  | 2.78 | 3.46E-02 |  | -3.11 | 4.47E-04 |  | 4.21 | 2.54E-03 |  | -1.67 | 1.67E-01 |
| Alanyl-Leucine | 5.74 | 2.11E-04 |  | -0.39 | 4.80E-01 |  | 4.78 | 1.72E-04 |  | -1.35 | 8.26E-04 |  | 4.70 | 2.50E-03 |  | -1.43 | 5.89E-01 |
| Tiglylglycine | -0.40 | 2.39E-02 |  | 0.19 | 9.50E-02 |  | -0.37 | 2.91E-02 |  | 0.22 | 5.17E-02 |  | -0.51 | 4.40E-03 |  | 0.08 | 3.64E-01 |
| ^a^Fold change was calculated as a binary logarithm of the average mass response (peak area) ratio between each group vs control group or between each group vs CRF group, where a positive value means that the average mass response of the metabolite in each group is larger than that in the control group or CRF group. ^b^The *p* value was calculated from Student’s t test. | | | | | | | | | | | | | | | | | |
